# Strong light–matter interactions: a new direction within chemistry

**DOI:** 10.1039/c8cs00193f

**Published:** 2019-01-21

**Authors:** Manuel Hertzog, Mao Wang, Jürgen Mony, Karl Börjesson

**Affiliations:** a University of Gothenburg , Department of Chemistry and Molecular Biology , Kemigården 4 , 41296 Gothenburg , Sweden . Email: karl.borjesson@gu.se

## Abstract

It is possible to modify the chemical and physical properties of molecules, not only through chemical modifications but also by coupling molecules strongly to light. More intriguingly, strong coupling between molecules and light is possible even without the presence of a photon. The phenomenon that makes this possible is called vacuum fluctuations, which is the finite zero point energy of the quantized electromagnetic field inside an optical cavity. The light–matter coupling, which can be as large as 1 eV (100 kJ mol^–1^), leads to the formation of new hybrid states, called polaritons. The formed hybrid states can be viewed as a linear combination of light (vacuum field) and matter (molecules), thus completely changing the energy landscape of the system. Using vacuum fluctuations, strong light–matter interactions have for instance been used to change chemical reactivity, charge conductivity, excited state relaxation pathways and rates of chemical reactions of organic molecules. In this review a brief history of the field is given, followed by a theoretical framework, methods of analysis, and a review of accomplishments. Finally, a personal reflection on the future perspectives and applications within this field is given.

## Abstract 2

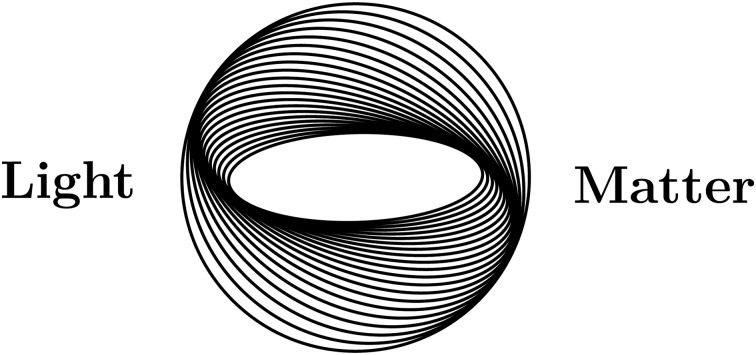
Strong light–matter coupling enables the possibility of changing the properties of molecules, without modifying their chemical structures, thus enabling a completely new way to study chemistry and explore materials.

## 1. Introduction

For centuries, scientists and philosophers have described matter as an independent and isolated component in nature. This reductionist vision has led to an approach to science where scientists analyzed phenomena based on the individual components of the system. Modifying the chemical and physical properties of molecules by chemical synthesis is therefore ubiquitous throughout science. In the classical hypothesis, the properties of a system are the trivial sum of the properties of their individual components. In 1972, Anderson published a seminal paper ‘More is different’ in which he described a distinction, in a conceptual way, between the properties of isolated constituents and the qualitative properties of a macroscopic system, a phenomenon defined as emergence.^1^ In molecular science, emergent properties are often due to intermolecular interactions. Exciton coupling is an example of how intermolecular interactions can affect molecular properties. Organic dyes can self-aggregate at high concentrations. If the distance between molecules is small enough, Coulomb interactions between the transition dipole moments on different molecules will be significant. This results in the resonant transfer of excitation energy from one molecule to another within the aggregate, *i.e.*, there is a delocalization of the excitation. The strength of the exciton–exciton coupling depends on the magnitude of the transition dipole moment, the relative orientation between monomers (transition dipole moments), and the intermolecular distance. The coupling can be regarded as “strong” if the time scale of excitation energy transfer is faster than other decay pathways of the molecule (spontaneous emission, dephasing, *etc.*).^2^ The system is now characterized with new eigenstates resulting from the coupling, giving a significant modification of the optical properties of the aggregate as compared to the isolated molecular case.^3^ Thus, the absorption spectrum of the aggregate is either red shifted (J-aggregates) or blue shifted (H-aggregates) depending on the relative orientation of the transition dipole moments between molecules in the aggregate.^4^

**Fig. 1:**
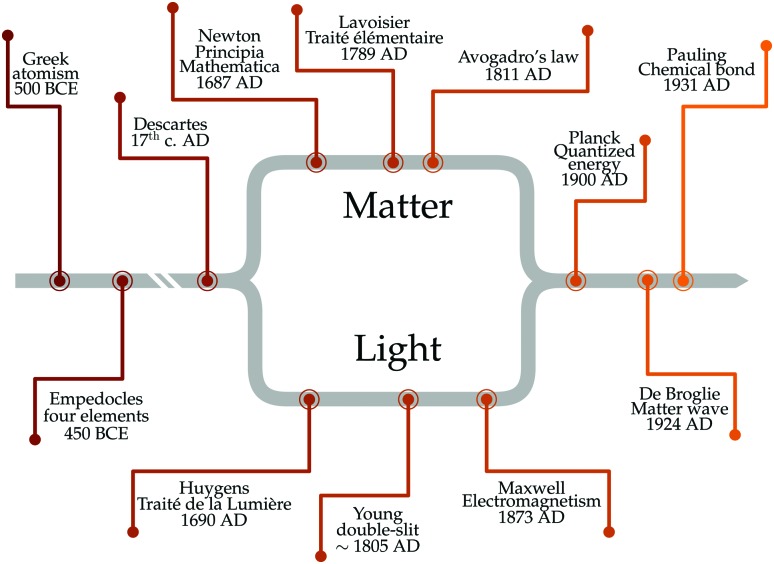
Historical evolution of the approach to explain light–matter interactions.

Similar to exciton coupling, molecular properties can be affected by other types of surroundings. Until the beginning of the 20th century, light and matter have been treated as different entities, with their own specific properties (Fig. 1). The development of quantum mechanics has enabled the theoretical description of the interaction between light-quanta and matter.^5^ An experimental demonstration of this interaction is, *e.g.* the modification of the radiative decay of a molecule in the vicinity of a metallic surface,^6^ or the Purcell effect.^7^ These two experiments have shown that molecules can be affected by the surrounding electromagnetic environment. However, only the radiative rate constants are affected and the system is in the so-called weak coupling regime. The first experimental demonstration of strong exciton–photon coupling (where not only radiative decay rates are affected, but new energy levels are formed) was reported by Yakovlev *et al.* in 1975.^8^ Likewise, in 1982, Pockrand *et al.* reported exciton–surface plasmon strong coupling of Langmuir–Blodgett monolayer assemblies on a silver surface.^9^ The theoretical description of these phenomena was first provided by Jaynes and Cummings^10^ and the coupling to surface plasmons by Agranovich and Malshukov.^11^ Over the following 20 years a tremendous interest arose in the field of strong coupling using inorganic semiconductors^12^–^14^ (quantum wells) or Rydberg atoms^15^–^18^ in high finesse Fabry–Pérot resonators at low temperature with remarkable realizations, *e.g.* polariton Bose–Einstein condensates,^19^–^24^ and superfluidity.^25^,^26^ The Nobel Prize in physics 2012 was awarded to Serge Haroche and David J. Wineland for their seminal work within strong light-atom coupling. Despite the considerable amount of literature published based on inorganic materials, as well as organic materials in microcavities in the weak coupling regime,^27^–^31^ strong exciton–photon coupling using organic molecules was only introduced theoretically^32^ by Agranovich *et al.* in 1997 and demonstrated experimentally one year later by Lidzey *et al.* in a pioneering paper.^33^ Following those milestones, it has been an ever-growing increase of interest in using organic molecules (Frenkel excitons) due to their large transition dipole moments providing a larger coupling strength as compared to inorganics (Wannier–Mott excitons).^34^–^44^ In fact, organic molecules enable, due to their large transition dipole moments and large binding energy, the possibility to explore the effect of strong coupling on the physical and chemical properties of organic molecules at room temperature.

## 2. Theoretical considerations when going into the strong coupling regime

**Fig. 2:**
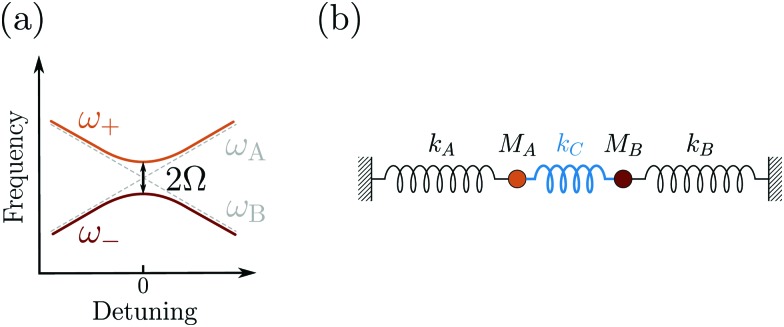
(a) Anticrossing behavior of two classical oscillators coupled together. Detuning is defined as the frequency difference between *ω*_A_ and *ω*_B_. (b) Schematic figure of two harmonic oscillators coupled together.

Despite its common usage, the term strong coupling is used in different disciplines to mean different things. We will introduce the basics of strong light-matter interactions by starting with the classical analogy of two coupled harmonic oscillators. When two harmonic oscillators are uncoupled, they behave independently. By this, they display their own measurable parameters (*e.g.* frequency). However, when the coupling between them is strong enough, the two oscillators start to exchange energy periodically and the system behaves as one single entity. Thus, the energy spectrum of the system is modified, leading to an avoided crossing (Fig. 2a). Let's consider two undamped harmonic oscillators with masses *M*_A_ and *M*_B_ and spring constants *k*_A_ and *k*_B_, coupled together with a spring constant *k*_C_ (Fig. 2b). Newton's second law describes the motion for the system:

1 *M*_A_*ẍ*_A_ + *k*_A_*x*_A_ + *k*_C_(*x*_A_ – *x*_B_) = 0

2 *M*_B_*ẍ*_B_ + *k*_B_*x*_B_ – *k*_C_(*x*_A_ – *x*_B_) = 0

Solving the differential equations yields two new normal modes:
3

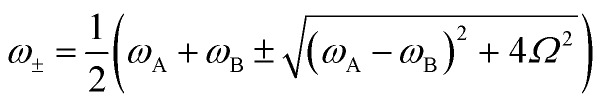

where *ω*_A_ and *ω*_B_ are the frequencies of the two oscillators, *Ω* is the frequency splitting and *ω*_±_ are the two new frequencies of the system. At resonance, *i.e.* when *ω*_A_ = *ω*_B_ ≡ *ω*, eqn (3) reduces to *ω*_±_ = *ω* ± *Ω*. The energy separation between the two new modes is called normal mode splitting (Fig. 2a). The phenomenon depends on the strength of the coupling spring constant *k*_C_ compared to the strength of the other spring constants. Eqn (1) and (2) do not take damping into account, which can be introduced with a frictional term (*γ*_A_*ẋ*_A_ in eqn (1), and *γ*_B_*ẋ*_B_ in eqn (2)). Two different regimes can be defined: the weak and the strong coupling regime. When *Ω* > (*γ*_A_/*m*_A_ + *γ*_B_/*m*_B_), *i.e.* the dissipation is smaller than the coupling strength, the system is in the strong coupling regime, leading to a shift of the eigenfrequencies and a characteristic frequency splitting (symmetric and anti-symmetric) of the system.^45^ These normal mode-splitting and avoided crossing phenomena are commonly observed in macroscopic systems, *e.g.* classical harmonic oscillators,^46^ acoustic waves^47^ or quantized mechanical oscillators.^48^ It is instructive to have the coupled oscillator model in mind when reading the following sections describing the underlying quantum aspects of strong light–matter interactions.

### 2.1 Vacuum fluctuations

Having defined what strong coupling means from a classical perspective, we will now move on to discuss the quantum description of the phenomenon. The classical description cannot explain all the signatures of strong coupling due to the presence of the vacuum field. Vacuum fluctuations, or zero-point energy, first appeared in Planck's second theory of blackbody radiation and Einstein's theory of molecular agitation at zero temperature in 1911 and 1913, respectively.^49^,^50^ In 1916, Nernst emphasized that the zero point energy exists in a field mode,^51^ which was later confirmed through the quantum theory of the electromagnetic field. Vacuum fluctuations are described as the ground state energy of the quantized electromagnetic field. The fundamental commutation relation [*x[combining circumflex]*,*p[combining circumflex]*] = *i*ℏ avoids simultaneous vanishing of the kinetic and potential energy, and the energy of the ground state is a compromise between those two energies due to the Heisenberg uncertainty relation. This pure quantum effect is mostly known to explain the Casimir effect^52^ and the Lamb shift in the atomic spectra of the hydrogen atom when particles interact with the fluctuations of the quantized vacuum field, leading to small energy shifts.^53^ Mulliken used vacuum fluctuations to explain the vibrational spectrum of Boron monoxide in 1924,^54^ a year before the quantum formalism. More recently, vacuum fluctuations were directly measured.^55^ In the case of an optical cavity (see Section 3.1 for definition), the strength of the vacuum electric field 
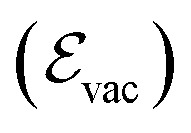
 is given by
4

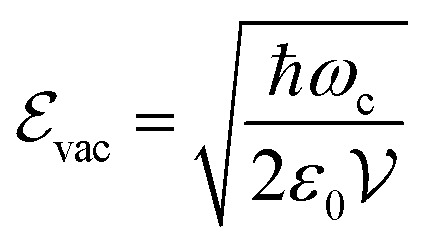

where *ω*_c_ is the cavity frequency, *ε*_0_ is the vacuum permittivity, and 
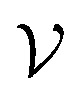
 is the mode volume of the cavity.

### 2.2 The interaction between light and matter

In order to describe light–matter interactions as coupled quantized harmonic oscillators, we need to make approximations. Many theoretical approaches rely on the long-wavelength limit, also called the dipole approximation, which assumes that the wavelength of the electromagnetic field is much larger than the molecular length scale. The coupling is therefore only described by the total transition dipole moment of the molecule and a uniform electric field. Furthermore, molecules remain with a high probability in their ground state, and the excited state energy level is modelled as a harmonically bound particle, excited by the light field. This approximation is called the Thomson–Lorentz model of the atom, and represents an atomic ensemble as a collection of oscillators. In addition, an electromagnetic field can also be described as a collection of independent oscillators.^56^ Before introducing a quantum description of strong coupling, we need to emphasize on the notion of the transition dipole moment. It is the key parameter that determines the light–matter interaction using a dipole–dipole interaction. The transition dipole moment (*d[combining right harpoon above]*) defines the strength of interaction that causes a transition between an initial state *Ψ*_i_ and a final state*Ψ*_f_:
5

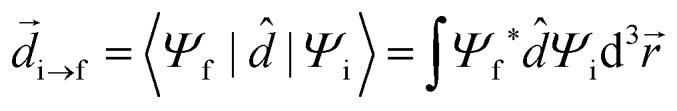

This explains why some molecules absorb and/or emit more than others. The coupling *V* between light and matter is described by a dipolar coupling:
6

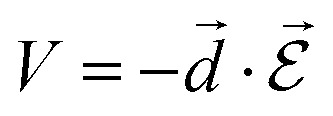

where 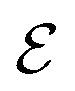 the electric field operator. The scalar product in eqn (6) implies that the relative orientation between *d[combining right harpoon above]* and 
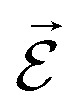
 is of importance as recently shown experimentally.^57^,^58^ Fermi's golden rule describes the transition rate (*Γ*) between two states:
7

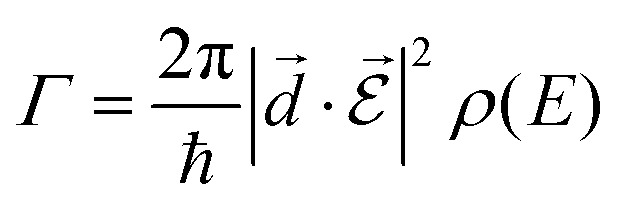

where *ρ*(*E*) is the density of states (DOS) at energy *E*. Fermi's golden rule states that the rate of spontaneous emission depends on the DOS of the coupled electromagnetic field. Thus, a change in the electromagnetic DOS can drastically change the rate of spontaneous emission, which is the so-called Purcell effect.^7^ An experimental demonstration of this effect at optical frequencies was first reported by D. J. Heinzen *et al.* in 1987.^59^ Inside a cavity, the spontaneous rate of emission is enhanced when the molecule is in resonance with a field mode, because the photon DOS is increased inside the cavity with respect to the free space DOS.

Intuitively, when a molecule emits inside a cavity, the photon is reflected by the mirrors and subsequently remains inside the cavity. Therefore, the probability of reabsorption by the molecule is enhanced. If this probability is higher than the probability of photon leakage through the mirror and the non-resonant decay rate of the molecule, then the system enters the strong coupling regime, which we will explore further in this review.

### 2.3 A quantum description of the strong coupling regime

In the following section, we will introduce the concept of strong coupling in the framework of cavity quantum electrodynamics (cQED), which describes the properties of a quantized electromagnetic field and an atomic system coupled together. A simple model in cQED consists of a two-level atom interacting with a single mode of the electromagnetic field. In this case, the system is described by the well-known Jaynes–Cummings Hamiltonian,^10^ which describes the system as the sum of the molecule, the electric field, and the molecule–field interaction within the rotating frame approximation:
8

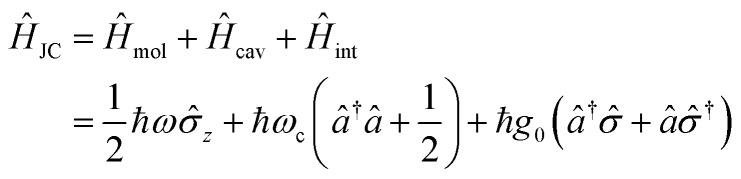

where *σ̂*_*z*_, *σ̂* and *σ̂*^†^ are Pauli matrices for inversion, raising and lowering, respectively, *â* and *â*^†^ are creation and annihilation operators for the field mode, *ω* and *ω*_c_ are the transition frequencies of the molecule and the cavity, respectively, and *g*_0_ is the magnitude of the light–matter coupling strength. In the interaction Hamiltonian (eqn (8)), the terms *â*^†^*σ̂* correspond to the transition from the ground state of the molecule to an excited state, simultaneously annihilating a photon in the cavity (*âσ̂*^†^ is the reverse process). This model can be extended to *N* molecules using the Tavis–Cummings Hamiltonian, by taking the limit of a large number of molecules and a small number of photons. This is done with the Holstein–Primakoff transformation,^60^ which changes the collective spin operator 
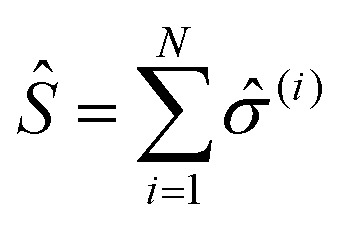
 into a bosonic operator, *b[combining circumflex]*, for each (*σ̂*_*z*_,*σ̂*,*σ̂*^†^). The collection of molecular two-level systems now acts like a giant quantum oscillator, which can be expressed with the following Hamiltonian:^61^,^62^

**Fig. 3:**
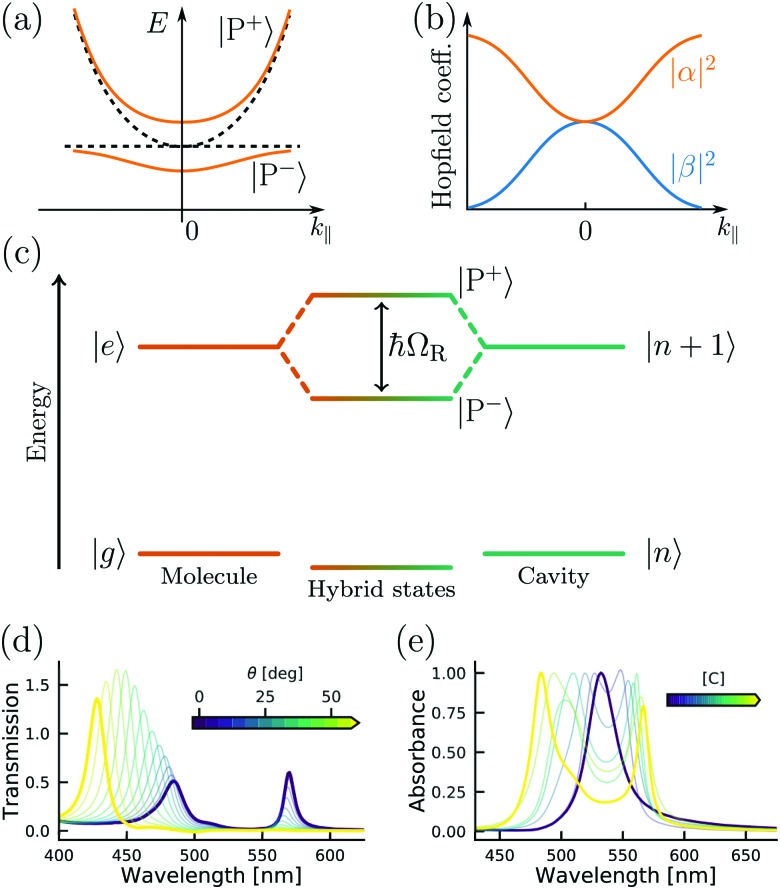
(a) Polariton dispersion at resonance and (b) corresponding Hopfield coefficients, where |*α*|^2^ + |*β*|^2^ = 1. (c) Jablonski diagram of a coupled molecule–cavity (electronic or vibrational transition) system showing the new hybrid polaritonic states with Rabi splitting ℏ*Ω*_R_. (d) Angle resolved transmission spectra of erythrosine B inside an optical cavity. (e) Absorption spectra of erythrosine B inside an optical cavity as a function of concentration (from 0.01 M to 0.54 M). Reproduced from ref. 184 with permission from Springer Nature, copyright 2018.

9

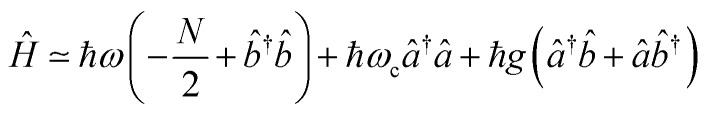

where 
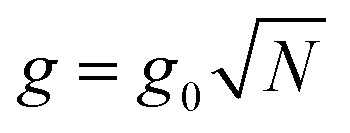
 is the collective coupling. The collective coupling is of fundamental importance and implies that the light–matter coupling strength is enhanced by a factor 
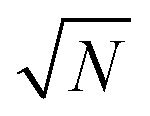
. Thus the molecular concentration is the relevant figure of merit for achieving a large *g*, since the coupling also depends on the field volume 
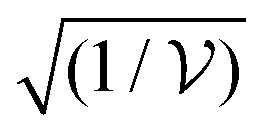
 (eqn (4) and Fig. 3e).

The Jaynes–Cummings Hamiltonian can be diagonalized *via* the Hopfield–Bogoliubov method yielding the two eigenstates of the system.^63^ The two eigenstates are linear combinations of light and matter, also called polaritonic states or polaritons. Polaritons are dressed states of the molecule–field system given by
10

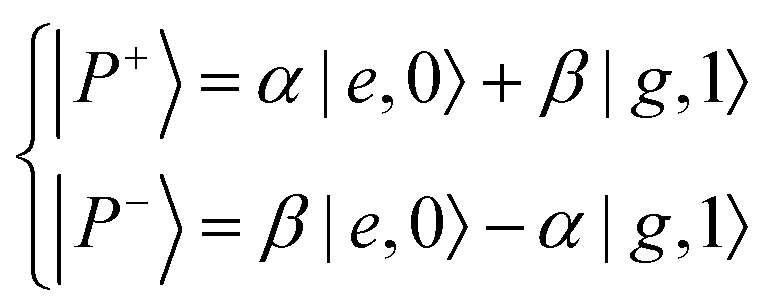

where |*g*〉, |, |*e*〉 represent, respectively, the ground and excited state of the molecule, and |0〉, |1〉 are the Fock states of the cavity ( represent, respectively, the ground and excited state of the molecule, and |0〉 represent, respectively, the ground and excited state of the molecule, and |0〉, |1〉 are the Fock states of the cavity (, |1〉 represent, respectively, the ground and excited state of the molecule, and |0〉, |1〉 are the Fock states of the cavity ( are the Fock states of the cavity (*i.e.* the number of photons). From eqn (8) and (9) we can extract the ratio between the optical and material character of the polariton, the Hopfield coefficient, defined as |*α*|^2^ and |*β*|^2^. At resonance, *i.e.* when the transition frequency of the molecule matches the frequency of the cavity, the polariton is a hybrid state of half-light and half-matter (Fig. 3b). We define the detuning of the cavity as red (or negative) when the frequency of the cavity is smaller than the molecular transition and as blue (positive) in the other case. The energy difference between the upper and lower polariton is called the vacuum Rabi-splitting (ℏ*Ω*_R_; Fig. 3c). It can be expressed as a function of coupling strength, *g*:
11

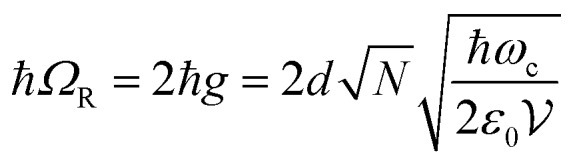

An important feature of polaritonic states is their dispersive behavior inherited from their photonic component. The cavity photon modes have a strong in-plane dispersion, and the energy for an optical cavity is
12

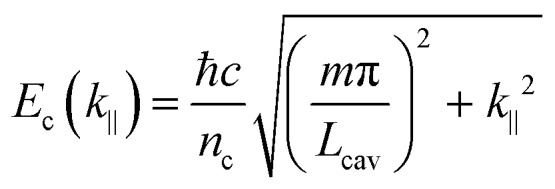

where *n*_c_ is the background refractive index, *c* the speed of light, *L*_cav_ the cavity thickness and *m* the cavity mode number. The in-plane wavevector *k*_‖_ is related to the wavelength and angle of incidence *θ* through
13

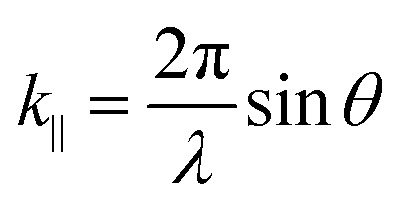

The energy of the exciton is wavevector independent, whereas the cavity photons show a strong in-plane dispersion. Thus, the resulting cavity polaritons also show a strong in-plane dispersion as shown in Fig. 3a and d. This strong angle dependence of the cavity mode energy implies that one can tune the resonance frequency by simply varying the angle of incidence when measuring.

This section has introduced the basics of the quantum description of strong light–matter interactions. One should remember the key parameters to increase the coupling: the transition dipole moment of the molecule, the number of molecules, the mode volume and the relative orientation between the transition dipole moment of the molecule and the electric field. However, the transition between weak and strong coupling is not clearly defined in the Jaynes–Cummings Hamiltonian. The next section describes the conditions to characterize a system in the strong coupling regime.

### 2.4 The strong coupling limit

At resonance the relative strength of the coupling is governed by three parameters: the photon decay rate of the cavity, *κ*, the non-resonant decay rate of the molecule, *γ*, and the coupling strength, *g*. These three parameters define, in the time domain, the dynamics of the system. When *g* ≪ (*κ*,*γ*) the system is in the weak coupling regime. Conversely, when *g* ≫ (*κ*,*γ*) the system is in the strong coupling regime. In the strong coupling regime, the light–matter interaction is faster than dissipation processes. Therefore, the molecule interacts coherently with the cavity and can emit and again absorb cavity photons several times before the cavity photon is lost. In other words, there is a delocalization of the energy in the system. Both *κ* and *γ* are related to experimental measurable parameters, that is the linewidth of the cavity and the linewidth of the molecular absorption band, respectively. Strong coupling occurs when the splitting (at resonance) is larger than the transmission linewidths, *i.e.* 2*g* > (*γ* + *κ*)/2.^64^,^65^ The transition between weak and strong coupling occurs when both polaritonic branches are spectroscopically resolved, *i.e.* the splitting (*Ω*_R_) must be larger than the full width at half maximum (FWHM) of the bare molecule absorption (Δ*ω*_X_) and the cavity mode (Δ*ω*_c_).
14

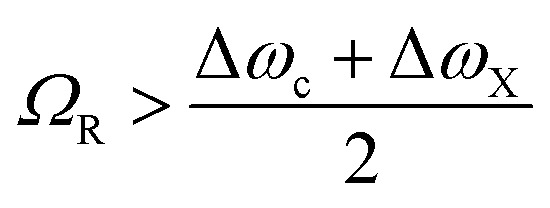

However, the aforementioned criterion is not sufficient to characterize the system as being in the strong coupling regime. In the weak coupling regime, splitting can for example occur because of inhomogeneous broadening,^66^ cavity induced transparency,^67^–^69^ or Fano resonance in plasmonic structures.^70^–^72^ Therefore, the dispersive nature of the polaritonic states should always be measured, as to visualize the anticrossing between the cavity mode and the exciton (Fig. 3d).

The relative strength of the coupling can be measured by the ratio between the frequency (or energy) of the cavity mode and the Rabi frequency (or energy). When this ratio is greater than 0.1 to 0.2 the system is in the so-called ultra-strong coupling (USC) regime.^26^ In the USC regime, the energy of the coupling is significant as compared to the energy of the molecular transition. This causes the rotating wave approximation to break down and needs to be described within the framework of the Dicke Hamiltonian.^73^,^74^Eqn (8) must therefore be modified to take anti-resonant terms into account.^75^ In the USC regime, new phenomena appear, *e.g.* photon blockades,^76^,^77^ superradiance,^78^ ground state modifications,^79^,^80^ and more recently using organic molecules^81^–^87^ to achieve ultra-efficient light emission.^88^–^90^

### 2.5 Modelisation of strong coupling phenomena

The following section will discuss the modelisation of hybrid light–matter systems. In order to fit and extract parameters from experimental data, the coupled harmonic oscillators (CHO) model is a common and simple approach.^91^ The CHO model treats the cavity and the exciton as coupled oscillators with a coupling element 1/2ℏ*Ω*_R_. This method has the advantage to be solvable analytically, yet captures most of the essential underlying physics. When dealing with one exciton and one cavity photon, the coupling is described by a 2 × 2 matrix Hamiltonian:
15

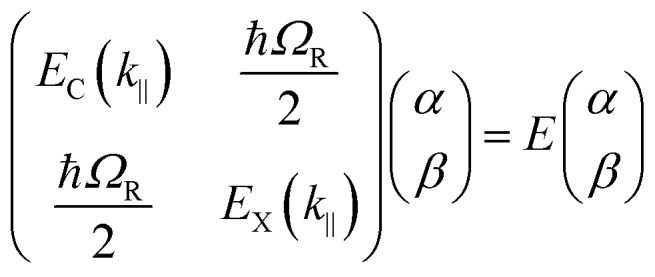

where *E*_C_ is the cavity energy, *E*_X_ is the exciton energy, and ℏ*Ω*_R_ is the Rabi-splitting. Diagonalization of this Hamiltonian leads to the eigenvalues of the Hamiltonian, which represent the polariton energies and in-plane dispersion (Fig. 3a).
16

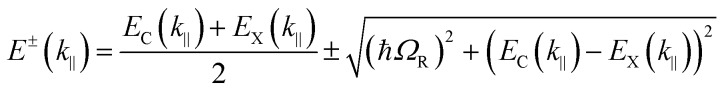

Eqn (15) (or (16)) can be used to fit the Rabi splitting (*E*_X_ and *E*_C_ are then fixed values taken from independent measurements), using spectra taken at different angles of incidence. Furthermore, one can extract the Hopfield coefficients through the eigenvectors:
17

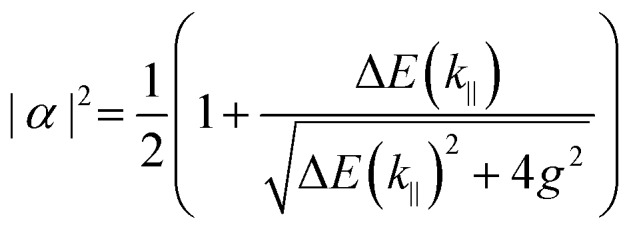



18

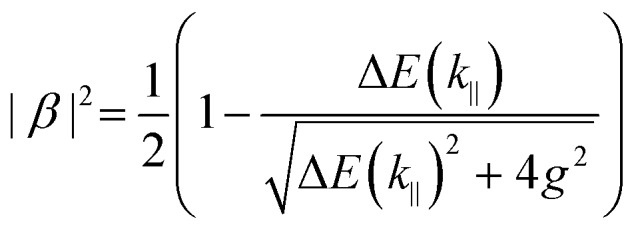

where Δ*E*(*k*_‖_) = *E*_X_(*k*_‖_) – *E*_C_(*k*_‖_). Eqn (15) can be extended to an *N* × *N* matrix to include more molecular transitions.

Another method to model experimental data is to use a pure electromagnetic approach based on Maxwell's equations. Both the Transfer Matrix Method (TMM) and the Finite-Difference Time-Domain method (FDTD) are widely used. The TMM approach can simulate the transmission/reflection spectra and the electrical field amplitude of a one-dimensional multi-layered structure. It calculates the electrical field in each layer and boundaries using Fresnel coefficients.^92^–^94^ This approach is very practical for Fabry–Pérot cavities. For plasmonic systems, the FDTD method is widely used and we refer interested readers to reviews from Gray for details on this method.^95^–^97^ Both these approaches take accurate account of the ‘light’ part of the strong coupling phenomenon but do not provide accurate information on the ‘matter’ part. While traditional approaches from quantum chemistry, such as the Born–Oppenheimer approximation, Hartree–Fock method,^98^ density functional theory^99^,^100^ (DFT), coupled-cluster theory^101^ or time dependent density functional theory^102^ (TDDFT), are able to describe the quantum nature of the matter they cannot account for the quantized nature of the electromagnetic field. To include the quantized electromagnetic field into TDDFT and have an *ab initio* description of light–matter interactions, quantum-electrodynamical density-functional theory (QEDFT) was introduced to unify light and matter approaches,^103^–^105^ and used, for example, to construct both electronic and photonic observables of an azulene molecule in an optical cavity.^106^ For more complete theoretical and computational descriptions of light–matter interactions, we refer interested readers to recent reviews.^107^–^109^

To conclude, simple analytical models are often used to describe strong light–matter interactions with good agreements to fit data and extract parameters (*e.g.* coupling energy, cavity energy, *etc.*). Recent years have seen a rapid development of *ab initio* description of strongly coupled light–matter systems, which can describe strong light–matter interactions more fundamentally.

## 3. Experimental methods to reach the strong coupling regime

The prerequisite condition to reach the strong coupling (SC) regime is a reversible exchange of energy, between an emitter (organic dye, quantum dot, *etc.*) and an electromagnetic mode of a cavity, on a faster timescale than energy dissipates from the system. The major function of the cavity is to confine the electromagnetic field, and thus enhance its interaction with the emitter. For the reversible exchange of energy to take place, the electromagnetic mode needs to be close to the resonant energy of a molecular transition (electronic or vibrational). In general, there are mainly two categories of cavities used in coupling organic molecules strongly to light: the optical cavity and the plasmonic cavity. In this section, we will go through the basic concepts of these two types of cavities, and discuss their properties in relation to strong coupling with organic materials.

### 3.1 The optical cavity

**Fig. 4:**
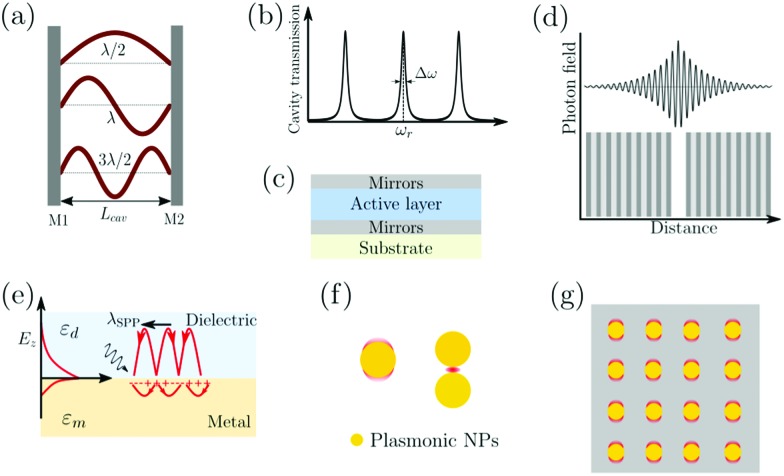
(a) The structure of a Fabry–Pérot cavity. The cavity length *L*_cav_ is equal to an integer number of intracavity half wavelengths (*λ*/2) between two mirrors (M1 and M2). (b) A typical transmission spectrum of a Fabry–Pérot cavity. The quality factor (*Q*-factor) is defined as the ratio between the resonant frequency (*ω*_r_) and the linewidth (*ω*) of the cavity mode. (c) A common structure of optical cavities used for strongly coupled organic materials, in which the active material is sandwiched between two mirrors. (d) A schematic of a typical DBR cavity. The DBR mirror contains multiple layers of alternating materials with high and low refractive index. The photon field penetrates a certain distance into the mirror. (e) Surface plasmon polaritons (SPPs) at a metal–dielectric interface. The electric field decays exponentially away from the interface. Because of this high spatial confinement, SPPs usually have a high local electromagnetic field intensity. (f) Typical plasmonic cavities including plasmonic nanoparticles, plasmonic gap and (g) surface plasmonic lattice.

An optical cavity containing an arrangement of mirrors forms a standing-wave resonator for electromagnetic waves.^110^ We shall restrict our discussion here to the simplest case, namely a planar cavity, also called Fabry–Pérot cavity (Fig. 4a). The cavity consists of two plane mirrors separated by an adjustable length. The mirrors are aligned parallel to each other so that light can bounce back and forth between the mirrors forming standing waves. The cavity is considered to be on-resonance when the light is in phase after one round trip, giving a maximum of the transmitted light through the cavity. Assuming no light penetration into the cavity mirrors, the resonance condition occurs when the cavity length, *L*_cav_, is equal to an integer number of intracavity half wavelengths (Fig. 4a):
19

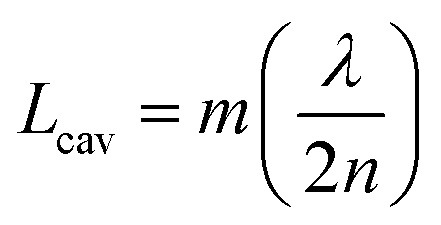

where *λ*, *n* and *m* are the wavelength of light, the refractive index of the material inside the cavity, and an integer number, respectively. The energy dissipation from a cavity (from absorption, scattering or leakage through the imperfect mirrors) is characterized by the quality factor (*Q*-factor):
20

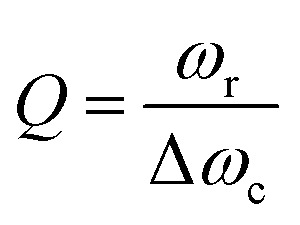

where *ω*_r_ and Δ*ω*_c_ are the resonant frequency and the linewidth (FWHM) of the cavity mode, respectively (Fig. 4b). Generally, a high *Q*-factor cavity is achieved by improving the reflectivity of the mirrors. For reaching the SC regime, the *Q*-factor should be as high as possible to reduce the energy dissipation from the cavity. However, there is no reason why the *Q*-factor should be much higher as compared to the linewidth of the molecular transition being coupled. This is because it is the total dissipation from the system (cavity + molecule) that should be slower than the exchange of energy between the molecules and the cavity. As a rule of thumb, a cavity with a FWHM roughly equal to the FWHM of the molecular transition being coupled offers a good compromise between *Q*-factor and transparency (the cavity needs to have a finite transparency to be probed).

A commonly used cavity structure for strong coupling with organic materials consists of (Fig. 4c) (1) a bottom mirror usually supported by a transparent substrate (*e.g.* glass, quartz, CaF_2_, ZnSe in the infrared, *etc.*); (2) an active layer, which normally consists of thin films of organic materials. It is common to disperse the chromophores into an inert matrix (*e.g.* polymer) for better film quality and/or to adjust the coupling strength (the coupling strength depends on the chromophore concentration); (3) a top mirror supported by the active layer. Thus, the thickness of the solid active layer governs the resonance frequency of the cavity (eqn (19)). It is also possible to make cavities using liquid medium as the active layer. However, this is technologically difficult for electronic transitions due to the nanometric scale of the cavity.^111^ For strong coupling to molecular vibrations, it is more easily achievable to have an active liquid medium, as it requires a cavity length at the micrometer scale.^112^

In the early reports of strong coupling with organic materials, distributed Bragg reflector (DBR) mirrors were mainly adopted because of the high quality factor (10^2^–10^5^) of the formed cavities.^33^,^113^,^114^ A typical DBR mirror contains multiple layers of alternating materials with high and low refractive indexes (Fig. 4d). The constructive interferences from the reflections from each layer add together to act as a high-quality reflector at a specified wavelength range. The *Q*-factor of the cavity depends on the number of pair repeats and the refractive index contrast between the two dielectric materials. The thickness (*l*) of each dielectric layer is about a quarter of the desired center wavelength (*λ̄*) of reflection:
21

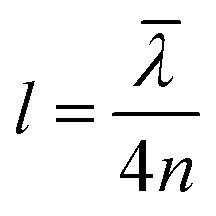

The resonant field of the DBR cavity reaches a maximum at the dielectric interfaces and there is a significant penetration of light into the mirrors. Materials such as SiO_2_ and LiF are often used as low refractive index layers, while the high index material usually is SiN_*x*_, HfO_2_, or TiO_2_.^115^ The inorganic oxide dielectric layers are usually deposited using plasma enhanced chemical vapor deposition (PECVD), which requires high substrate temperatures, preventing deposition directly on organic materials. For top DBR mirrors, LiF and SiO_2_, which can be deposited by high vacuum thermal evaporation without damaging the underlying organic film, are often the first layers to be deposited.

The fabrication of DBR mirrors requires advanced deposition methods like chemical vapor/atomic layer deposition, which are commonly expensive and time demanding. In contrast, metallic mirrors are much easier to fabricate. Metallic mirror based cavities have, therefore, become more popular in recent years. Ag, Al and Au are the common metals used as mirrors because of their high reflectivity and easy fabrication process. Metals can conveniently be deposited on the surface of organic materials with low or no damage using DC magnetic sputtering or thermal evaporation. However, in our experience it is easier to make high quality mirrors made of Au and Ag as compared to Al, when depositing on very soft organic materials. Metallic mirror based microcavities have a relatively low *Q*-factor (10–100), which is limited by the intrinsic reflectivity (*R*) of the metals (in the visible range: *R*_Al_ = 88–92%, *R*_Ag_ = 95–99% and *R*_Au_ = 98–99% at *λ* > 550 nm). However, since molecular transitions in the condensed phase are broad (high rate of energy dissipation), the small *Q*-factor of metallic mirror based cavities does not pose a limitation when coupling organic molecules. The plasmonic effects from the metal are often ignored when making metal mirror based cavities. As the active materials are in direct contact with the metal mirror, the plasmon from the metal can cause distorted mode dispersion curves and lead to inaccurate evaluation of SC's effect on material properties.^116^ A thin protection layer is therefore advised to be inserted between the metal mirror and the active material, as to avoid plasmonic effects from the metal mirrors.

The boundary conditions for reflection of light at a metal imply that the optical field intensity is nearly zero at the mirrors. Thus, the penetration depth of light into the metal mirror (on the order of 10 nanometers) is much smaller as compared to DBR mirrors (on the order of hundreds of nanometers).^117^ The effective cavity volume is therefore much larger when using metallic as compared to DBR mirrors. The coupling strength between light and matter depends on the number of molecules in the cavity mode volume (eqn (11)), and a larger collective Rabi splitting can therefore be achieved when using metallic as compared to DBR mirrors, because the larger available space in the mode volume allows for a larger number of molecules in the cavity volume. Today, the metal mirror based Fabry–Pérot cavity is probably the most extensively used system to explore the collective behavior of molecular materials in the strong coupling regime.

### 3.2 Plasmonic cavities

Surface plasmons (SPs) are coherent delocalised electron oscillations that exist at the interface between any two materials where the real part of the dielectric function changes sign across the interface (*e.g.* a metal–dielectric interface, Fig. 4e). The charge motion of a surface plasmon can create electromagnetic fields outside (as well as inside) the metal. The surface plasmon polaritons (SPPs) are hybrid modes between light and the SP localised around the metal–dielectric interface.^118^,^119^ Because of the confinement effect from SPs, a SPP has a shorter wavelength than the incident free-space light.^120^ The confined volume of SPPs usually leads to a high local electromagnetic (EM) field intensity, which decays exponentially away from the metal/dielectric interface. SPPs preserve their bosonic photonic character, acting as confined photons. When coupled to organic molecules, the small volume as well as the high intensity of the EM field from SPPs allows reaching the strong coupling regime using a few or even a single molecule.^121^,^122^ However, it should be noted that the coupling strength depends on the number of emitters (*N*) and the cavity volume (*V*), and thus it is the molar concentration 
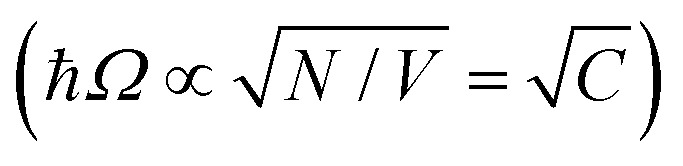
 in the mode volume that dictates the coupling strength, regardless of which type of cavity is used.

The metal surface can be structured in order to tune the resonance frequency and further confine the cavity volume.^121^ These structures are generally divided into three categories (Fig. 4f and g): (1) plasmonic nanoparticles or nanovoids (including spheres, disks, rods, shells, *etc.*) having a length scale of tens of nanometers, which can confine the electric field in all three dimensions and act as localized surface plasmons; (2) plasmonic gaps such as bowtie nanoantennas, nanorod dimers, and the gap between nanoparticles and planar metal surfaces. When two metal surfaces are located at a close distance (a couple of nanometers or even smaller) a plasmonic gap is formed. The electric field inside the gap is significantly enhanced because of the extreme confinement of the field volume; (3) plasmonic lattices, which are periodic arrays of nanoparticles.^123^ They support the hybridization of localized plasmonic resonances and the diffractive modes of the array, which are referred to as surface lattice resonances (SLRs). It offers advantages like a high quality factor due to Fano interference of the plasmon and the diffractive modes, and dispersion of the modes due to the two-dimensional geometry.

In summary, owing to the fast development of nanotechnology and nanofabrication in the past few decades, it is possible to fabricate almost any kind of optical or plasmonic cavity imaginable. Optical cavities offer ease of fabrication of large area systems, suitable when probing collective strong coupling using spectroscopy. Plasmonic cavities on the other hand are nanoscale objects often requiring microscopy techniques to be probed, but offer the possibility of direct contact with the formed polaritons due to the open nature of the cavity. In conclusion, the fabrication of cavities will not act as a fundamental obstacle for exploring strong light–matter coupling in the field of chemistry and materials science.

## 4. Polaritonic chemistry

Although strong light–matter coupling has been known for about 50 years, and strong coupling to organic molecules for 20 years, it is only during the last 10 years that the chemical aspects of the coupling have started to be explored. The realization that the chemical properties of molecules can be changed in a reversible manner by strong coupling to light is therefore a relatively new one. Strong coupling of molecules to the vacuum field provides a way of modifying molecular properties without structural modifications, offering a completely new tool to tweak matter. Considering changes of molecular properties under strong coupling, two different aspects need to be taken into account. Firstly, the energetics of the system is changed. The potential energy surface (PES) of the coupled state can be changed with up to 0.5 eV (∼48 kJ mol^–1^). Thus, it is not only possible, but also likely, that intramolecular processes that happen in the excited state, such as emission of a photon or photoisomerisation, are affected. Furthermore, it is not only the energy of the coupled excited state that is altered by the coupling, but also the ground state is perturbed. The perturbation of the energy of the ground state is not as large as for the excited state. However, for two molecules in rapid equilibrium where one of them is coupled to the vacuum field and the other is not, the change of the energy of the ground state is enough to change the equilibrium. Secondly, strong coupling is a collective phenomenon. Classically, the transition dipole moments on different molecules in the strong coupling regime can be viewed as being oscillating in phase. Energy is emitted from one molecule, bouncing back and forth in the cavity, and then reabsorbed by another molecule. Information on the phase of the transition dipole moment is therefore transferred between molecules. Quantum mechanically, the polaritonic states can instead be viewed as delocalized over a vast number of molecules. Communication in-between molecules far apart is hence increased, which is of great importance when considering applications such as charge or energy transfer. It should be noted that these two effects are only present inside the cavity. The changes of the molecules are thus reversible; if a molecule is removed from the cavity, the properties of the molecule will be the same as before being placed inside the cavity. In theory, it is consequently possible to use a cavity as a catalyst (one should keep in mind that the standard Gibbs free energy of the reactant or product is modified, see Section 4.3, the term catalyst is therefore not completely accurate as per definition), allowing a chemical modification to be performed inside the cavity, which afterwards is removed.

Strong light–matter interactions have been demonstrated in many different systems, including molecules coupled to single plasmonic cavities,^124^–^129^ and a single molecule coupled to a single plasmonic structure.^122^ In addition to organic molecules and inorganic structures in the solid state, some more exotic materials have shown hybridization with light. For example, different kinds of polymers,^23^,^87^,^130^–^135^ molecules in liquid phases,^85^,^112^,^136^–^138^ organic liquid crystals,^57^,^139^,^140^ organic crystals,^44^,^141^,^142^ quantum dots,^42^,^143^–^145^ nanocrystals in various geometries,^124^–^126^,^146^–^156^ nano-wires,^157^–^161^ nanographene,^162^ proteins,^163^–^165^ light-harvesting complexes,^166^ and photosynthetic bacteria have been hybridized with light.^167^,^168^ In the following sections, a review of accomplishments within the strong coupling regime is made. We will start with strong coupling to electronically excited states (Section 4.1). The section will cover the properties of polariton emission, changes in excited state quantum yields, and the effect on photochemical reactions. This is followed by sections on vibrational strong coupling (Section 4.2) and the effects on the ground state (Section 4.3). Finally, intermolecular communications (Section 4.4) and energy transfer (Section 4.5) in the strong coupling regime are covered.

### 4.1 The effect of the electronically excited state

After photoexcitation of an organic molecule to an electronically excited state, the energy is generally rapidly relaxed to the first excited singlet state (S_1_). Due to this fast intramolecular relaxation, the photochemistry and physics of organic molecules is governed by the properties of the S_1_ state. In the strong coupling regime, the hybridized molecular state is replaced by two hybrid light–matter states, in energy located around the original molecular state. If the coupled molecular state also is the first excited singlet state (S_1_), then the photochemistry and/or photophysics of the system can be dramatically changed.

#### 4.1.1 Emission from polaritonic states

**Fig. 5:**
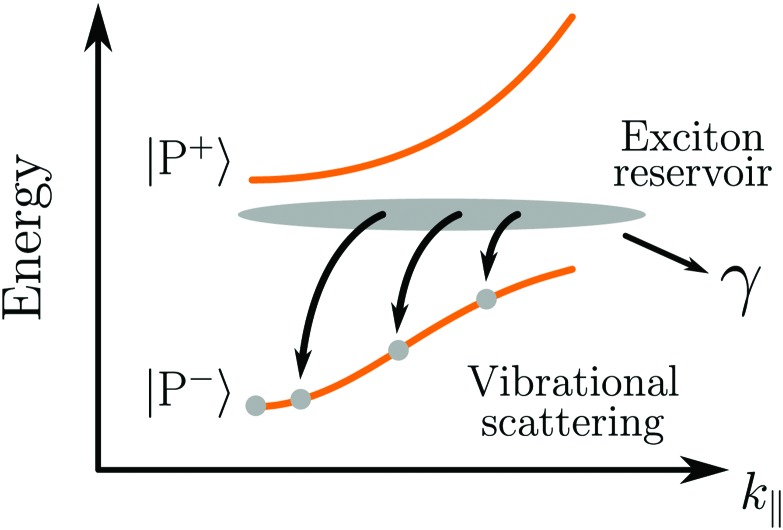
Vibrationally assisted relaxation processes from the exciton reservoir to the lower polaritonic branch. *γ* represents the dissipation of energy from the exciton to the environment (exciton decay, bimolecular quenching, *etc.*).

In the strong coupling regime, *N* molecules and one cavity mode hybridize, forming *N* + 1 new hybrid states.^169^ Two of these new states are the upper and lower polaritons, which are separated in energy by the Rabi splitting. The other *N* – 1 states are optically inactive states, located at *E*_X_, having more or less no cavity contribution (being orthogonal to the two polaritons), and are often referred to as the exciton reservoir or exciton bath. However, these dark states have an important influence on the relaxation pathways in a strongly coupled system. After initial excitation to the upper polaritonic state, relaxation down to the exciton reservoir (at the time scale of 150 fs)^170^ is faster than the emissive process. The reason for the fast relaxation is the large (*N* – 1) density of states in the exciton reservoir, which according to Fermi's golden rule results in a fast decay. Only one lower polaritonic state exists, and thus, the density of states is low and the rate of relaxation from the exciton reservoir down to the lower polaritonic state is therefore slow. In fact, the rate of relaxation down to the lower polaritonic state is expected to be much slower as compared to the rate of emission from the lower polaritonic state (which should be very fast due to the photonic contribution to that state).^22^ The observed polariton lifetime can therefore be seen as being dominated by the rate of relaxation from the exciton reservoir down to the lower polariton (Fig. 5).^171^,^172^ Experimentally, the observed polariton lifetime is in some cases even exceeding the lifetime of the uncoupled molecule.^173^,^174^ However, some experimental observations cannot be explained by an exciton reservoir model, but instead suggest a longer intrinsic lifetime of P^–^.^173^ Electronically excited states of molecules contain vibrational energy levels. These vibrations have been shown to assist in the relaxation from the exciton reservoir to the lower polariton.^172^ The exciton reservoir theory is indirectly confirmed by the experimental observation that the lifetime of emission, in contrast to the envelope of emission, is not showing a dispersive behavior, even though the photonic/excitonic ratio of the lower polariton is angle dependent (eqn (17) and (18)).^175^ Besides emission from the lower polaritonic state, excitonic emission (and absorption) is still observed in many cases but at a much lower relative intensity.^176^ Also emission from the upper polariton is observed in a few cases,^177^*e.g.* in the case of a small Rabi splitting where the thermal energy was high enough to repopulate the upper polariton^178^ or by a radiative pumping mechanism dominant at low temperatures.^170^

As already mentioned, the emission from a strongly coupled light–matter system has a few characteristic properties. The envelope of emission from the lower polariton shows a dispersive behavior, in which the emission is blue shifted with increasing angle. This is explained by the fact that the lower polariton is a linear combination of a photonic mode and a molecular exciton. With increasing angle the energy of the photonic mode increases (eqn (12)), causing a blue shifted emission.^179^ In addition, also the intensity of emission shows a dispersive character, which depends on the relative rates of P^–^ emission and relaxation of the exciton reservoir (Fig. 5). Another characteristic feature of polariton emission is narrowing of the emission bandwidth as compared to the corresponding exciton emission. It was theoretically proposed for inorganic semiconductors (quantum wells) in 1996 showing the narrow bandwidth for a hybrid state with an equal photonic/excitonic contribution to the lower polariton.^180^ Another interesting property that arises in the strong coupling regime is spatial coherence of the emission, which we will explain in more detail in Section 4.4.

In conclusion, polariton emission is dispersive by nature, from both photon energy and photon intensity perspective. It has a narrow bandwidth and emission from molecules micrometers apart can be coherent. The lifetime of emission is governed not by the intrinsic lifetime of the lower polariton, but rather by the rate of populating the lower polariton from dark decoherent states.

#### 4.1.2 Quantum yield of photophysical processes

The quantum yield (QY) of a photophysical process is defined as the number of occurred events divided by the number of possible events. The quantum yield of emission is thus defined as the number of emitted photons divided by the number of absorbed photons. For an optical cavity containing squaraine in a NPB (*N*,*N*′-di(1-naphthyl)-*N*,*N*′-diphenyl-(1,1′-biphenyl)-4,4′-diamine) matrix, the photoluminescence quantum yield increased from 0.01% to 0.03% when going into the strong coupling regime. When the system was excited resonantly at the energy of the upper polariton, it even reached a photoluminescence quantum yield of 0.2%.^181^ BODIPY-Br strongly coupled to an optical microcavity on the other hand showed similar emission quantum yield in the weak and strong coupling regime.^182^ In another study, the position of J-aggregated TDBC ((5,6-dichloro-2[3-[5,6-dichloro-1-ethyl-3-(3-sulfopropyl)-2(3*H*)-benzimidazolidene]-1-propenyl]-1-ethyl-3-(3-sulfopropyl)benzimidazolium hydroxide, inner salt, sodium salt)) inside a Fabry–Pérot cavity was considered. The photoluminescence quantum yield for molecules positioned at the antinode of the *λ*/2 and *λ* mode was determined to be 0.8% and 1%, respectively. Both values are lower as compared to a bare film having a QY of 2%.^173^ Not only investigations on the emission quantum yield from solid-state Fabry–Pérot cavities exist, but also liquid cavities have been explored. The emission quantum yield of Chlorin e6 dissolved in dimethylformamide was determined using low (weak coupling regime) or high (strong coupling regime) concentrations of Ce6.^111^ The quantum yield was determined relative to a dilute solution of Ce6. The QYs in the weak and strong coupling regime were 15% and 3%, respectively. It was concluded in the study that the difference in the emission QY between the weak and strong coupling regime is due to self-quenching at high concentrations rather than an effect of strong coupling.

A competing process of fluorescence is intersystem crossing (ISC) where the excited singlet state passes to an excited triplet state. The triplet state is lower in energy than the singlet state and further transition to the singlet ground state is quantum mechanically forbidden. Heavy atoms enhance the rate of ISC because of the increased spin–orbit coupling. The first investigations on how the rate of ISC is affected by strong coupling were made using a platinum porphyrin (2,3,7,8,12,13,17,18-octaethyl-21*H*,23*H*-porphyrin platinum(ii)) inside an optical cavity.^183^ The system showed a green polaritonic emission as well as red emission due to phosphorescence from uncoupled molecules. At resonant excitation of the polariton, the uncoupled phosphorescence as well as the polaritonic emission was observed. Furthermore, comparing the ratio of polaritonic to phosphorescent emission for resonant and non-resonant excitation, no significant changes were observed, leading to the conclusion that ISC from the polariton itself to the triplet excited state is indeed possible.

**Fig. 6:**
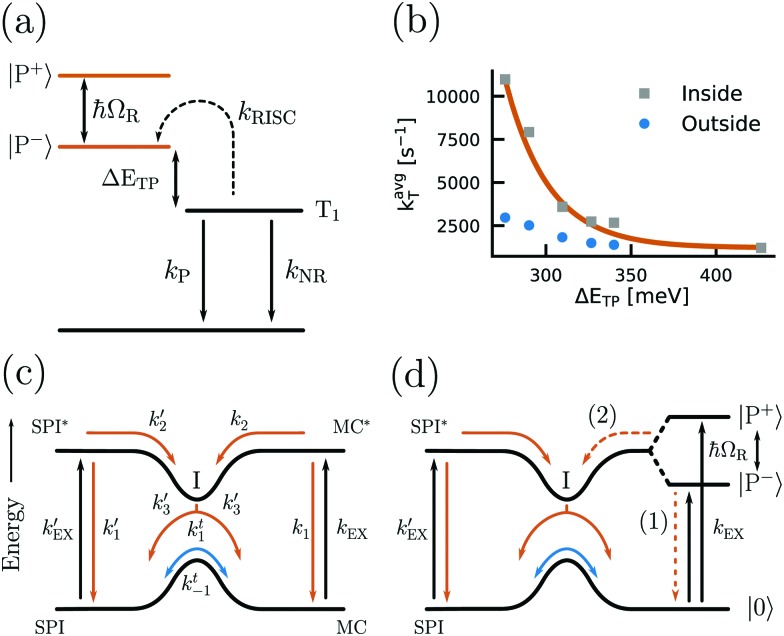
(a) Energy diagram showing the kinetics of triplet state depopulation by reversed intersystem crossing (*k*_RISC_), phosphorescence (*k*_P_) and non-radiative decay (*k*_NR_), and the energy difference (Δ*E*_TP_) between the lower polariton (P^–^) and the triplet state (T_1_). (b) Plot of the average triplet state depopulation rate (*k*avgT) as a function of the energy difference *E*_TP_. The rates outside and inside of a cavity are compared. Reproduced from ref. 184 with permission from Springer Nature, copyright 2018. (c and d) Diagrams of the energy landscape connecting the two isomers spiropyran (SPI) and merocyanine (MP) with different rates of excitation and relaxation. The case of modified MC states by strong coupling (d) is compared to the unmodified case (c) and the introduction of an energy barrier from the lower polariton (P^–^) to the ground state of SPI (process (2)) is clearly seen. Reproduced from ref. 185 with permission from John Wiley and Sons, copyright 2012.

The Rabi splitting depends on the transition dipole moment associated with the state being coupled (eqn (11)). Strong coupling can therefore be used to modify the relative energy differences between excited states. The rate of reversed intersystem crossing (RISC) depends on the energy difference between the first excited triplet state and the first excited singlet state. The first excited singlet state of erythrosine B has been strongly coupled to a cavity, leaving the energy of the triplet state unperturbed.^184^ By comparing the phosphorescence lifetime of the system in the strong coupling regime with one in the weak coupling regime it was found that the rate of RISC is enhanced by a factor of four due to a reduced energy gap between the lower polariton and the triplet state (Fig. 6a and b).

In conclusion, various photophysical properties have been shown to be modified in the strong coupling regime. Depending on the system, emission quantum yields can be either suppressed or enhanced. In addition, intersystem crossing from polaritonic states to an uncoupled triplet state is possible, and the rate of reversed intersystem crossing can be enhanced. Thus, strong coupling offers the possibility to tune the rates and yields of excited state relaxation processes.

#### 4.1.3 Photochemical reactions

Strong coupling completely modifies the potential energy surface of the excited state. Two new polaritonic states, having higher and lower energy, replace the uncoupled molecular excited state. These new states, not surprisingly, influence light-driven reactions.

The kinetics of the photoisomerization of spiropyran to merocyanine has been investigated in a Fabry–Pérot cavity.^81^,^185^ It was observed that the reaction rate decreased in the strong coupling regime. The reason for this observation is that the lower polariton has a lower energy as compared to the uncoupled molecular state. To photoisomerize, the system now needs to overcome a larger transition state energy, thus slowing down the reaction (Fig. 6c and d). This observation shows that it is possible to alter the chemical energy landscape in terms of reaction rate and yield by the introduction of molecules into the strong coupling regime. Also, plasmonic arrays have been used for photoswitchable strong coupling.^186^

For theoretical considerations of polaritonic chemistry, the concept of polaritonic potential energy surfaces (PoPES) was introduced by Feist *et al.* in 2017. PoPES is a combination of potential energy surfaces (PES) and quantum electrodynamics (QED), taking the electronic, nuclear and photonic degrees of freedom fully into account.^187^,^188^ The concept was applied to study the suppression of photoisomerization reactions. Three stabilising effects of the initial molecule towards the photoisomerization were found. First of all, the reaction barrier increases due to the lower energy level of the lower polariton compared to the bare molecular excited state. In addition, the Franck–Condon factor for a large number of molecules becomes approximately diagonal, suppressing the transition from the overall ground state to the vibronically excited states of the lower polariton. Finally, the PoPES has a narrow avoided crossing at the position where the hybridized collective state is changing to the excited singlet state of a single molecule, which is stabilizing the lower polariton even further.^189^ In another consideration, PoPES was used to demonstrate that only one photon is needed to photochemically convert many molecules. This is because molecules hybridized to a single photon in the strong coupling regime behave like a single polaritonic supermolecule, thus enabling apparent photoisomerization quantum yields higher than 1.^190^

Not only the modified energy landscape of a molecule in the strong coupling regime influences its photochemical reactions, but also the changed rates between excited states are of importance. The photobleaching of the J-aggregated TDBC has been shown to be suppressed when strongly coupled to a silver nanoprism. Photobleaching is a photooxidative process that contains several steps. In the first step, the photoexcited molecule undergoes intersystem crossing from the singlet to the triplet excited state. Molecules in the triplet state can react with triplet oxygen, generating singlet oxygen, which is unstable and therefore chemically damages the fluorophore. However, in the strong coupling regime the polaritonic lifetime is shorter, thus reducing the yield of ISC. Therefore, the system is unable to populate the triplet state efficiently and photobleaching is avoided.^191^

Strong coupling can theoretically also modify the nuclear dynamics of individual molecules in an ensemble.^192^ The time scale of energy exchange between electronically excited states can be faster than the time scale associated with nuclear motions, leading to a suppression of nuclei reorganization upon excitation—so called polaron decoupling. This phenomenon can be utilized to control the rate of electron transfer. The rate of intramolecular electron transfer was calculated to be enhanced by orders of magnitude. Besides intramolecular electron transfer, it was argued that the cavity-induced polaron decoupling has the potential to control other electron or energy transfer processes that involve nuclear arrangements in excited electronic states, which will be discussed in more detail in Section 4.5.

### 4.2 SC to the vibrationally excited state

**Fig. 7:**
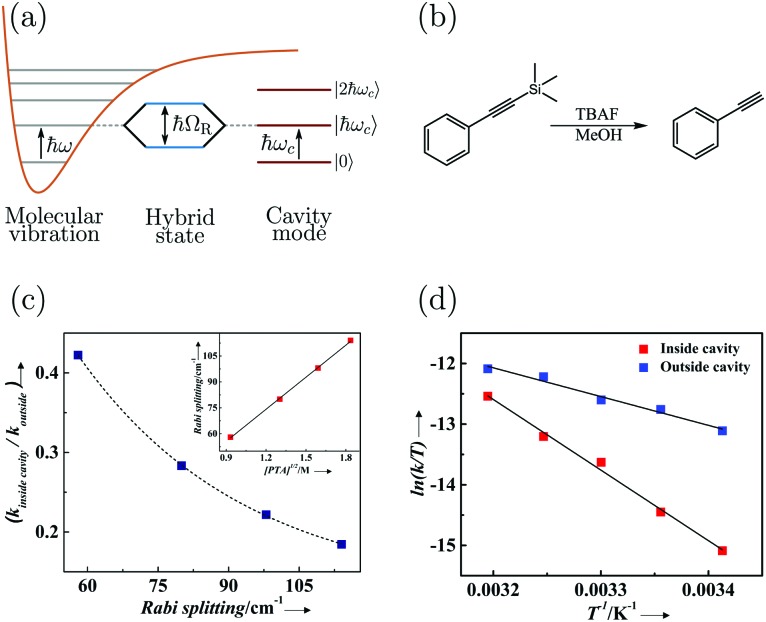
(a) Schematic picture of strong coupling between a molecular vibration and a cavity mode. (b) The silane deprotection reaction of 1-phenyl-2-trimethylsilylacetylene studied in ref. 189. TBAF refers to tetra-*n*-butylammonium fluoride. (c) The ratio of the reaction rate inside (VSC) and outside of the cavity as a function of Rabi splitting. The inset shows the linear dependence of the Rabi splitting on the square root of the concentration of PTA (1-phenyl-2-trimethylsilylacetylene). (d) The reaction rate ([PTA] = 2.53 M giving a Rabi splitting of 98 cm^–1^) as a function of temperature for reactions inside (VSC) and outside of the cavity. Reproduced from ref. 197 with permission from John Wiley and Sons, copyright 2016.

Strong coupling is not limited to electronic transitions. When the cavity field is in resonance with a vibrational transition, it is expected that the vibrational level will split into two polaritonic modes, separated by the vibrational Rabi splitting (Fig. 7a). Conceptually, there is no difference between strong coupling of light to electronic and vibrational transitions. However, the effects on the molecule by the coupling are different because electronic transitions are “high-energy” (from ∼1 eV to ∼3 eV) transitions involving electrons delocalized over the entire molecule, whereas vibrations are localized and have lower energy (from ∼37 meV (300 cm^–1^) to ∼434 meV (3500 cm^–1^)). The first example of vibrational strong coupling (VSC) was reported in 2015.^131^ The (C=O) bond of polyvinyl acetate located at 1740 cm^–1^ (215 meV) was coupled to the vacuum field using a ∼2 μm thick Fabry–Pérot cavity. Due to the extremely high concentration of (C=O) bonds inside the cavity, the collective Rabi splitting reached 170 cm^–1^ (21 meV), exceeding the dissipation rate of the system. Using transmission spectroscopy, an avoided crossing between the upper and lower polaritons was clearly observed as the angle of incidence was increased. Later in the same year, liquid-phase VSC for a variety of functional groups (C=O, C=C) of different molecules was demonstrated.^112^ So far, a variety of VSC systems using molecules in liquid and solid states have been reported,^57^,^85^,^130^,^134^–^138^,^164^,^193^–^198^ including polymers,^130^,^131^ organometallic complexes^137^ and protein vibrational modes.^164^ Even liquid crystals have been strongly coupled to an electromagnetic mode inside a combined cavity/liquid crystal cell.^57^ A brushed polyimide layer was included in the cavity, providing a boundary condition for the liquid crystal to orient. By applying a voltage over the cell, the liquid crystal was reorienting, changing the magnitude of light–matter interactions and thus providing a voltage control of the Rabi splitting in the strong coupling regime. Moreover, strong coupling between molecular vibrations and surface plasmon polaritons has been achieved,^198^ and a quantum mechanical formalism to treat interactions between molecular vibrations and an electromagnetic mode in the strong coupling regime has been developed.^169^

By changing the vibrational energy of a chemical bond, the ground potential energy surface of chemical reactions involving this bond is perturbed. In 2016, the change of ground-state chemical reactivity under vibrational coupling to the vacuum EM field was demonstrated.^197^ By strongly coupling a vibration along a carbon–silyl bond of a simple alkynylsilane (1-phenyl-2-trimethylsilylacetylene) to the vacuum EM field of a resonant infrared (IR) microfluidic cavity, the rate of Si–C bond breakage was affected (Fig. 7b). The reaction rate decreased by a factor of up to 5.5 when the Si–C vibrational stretching mode of the reactant was strongly coupled. The relative change in the reaction rate outside a cavity (uncoupled condition) and inside a cavity (strongly coupled) was found to depend on the Rabi splitting energy, which was controlled by the concentration of the reactant 
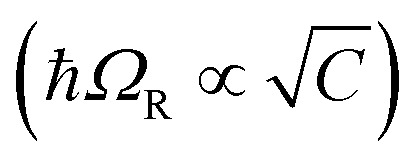
 (Fig. 7c). The temperature's effect on the reaction rate was further studied and the thermodynamic parameters associated with the transition state were extracted using the Eyring equation (Fig. 7d):
22

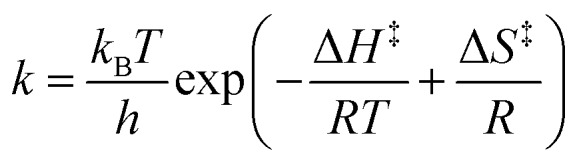

where *H* is the enthalpy and *S* is the entropy of activation. The change of *H* and *S* was found to be significant. At a Rabi splitting of 98 cm^–1^, *H* increased from 39 to 96 kJ mol^–1^, while *S* increased from –171 to 7.4 J K^–1^ mol^–1^. The change of the sign of the entropy and the large change of the enthalpy of activation indicate that the transition state was modified from an associative to a dissociative type. However, in the report, the bond strength of Si–C in the reaction was about 3.30 eV and the Rabi splitting was about 90 cm^–1^ (12 meV), which was almost negligible compared to the bond strength itself (0.36%). Although the VSC does not affect the bond strength so much, it still has a significant effect on the chemical reaction. The exact mechanism is still not clear, and more studies are needed to uncover the effects of VSC on chemical reactions. Although the reaction in the report is one of the simplest chemical reactions, it opens a new way to control chemistry. It is easily envisioned that strong coupling could act as a promoter of chemical reactions. However, it requires an increase instead of decrease of reaction rate in the strong coupling regime, and preliminary results suggest on this possibility.^199^

### 4.3 The effect of strong coupling on the ground state

The energetic landscape of a molecule changes when entering the strong coupling regime. However, not only the energy of the excited state changes by the formation of two new hybrid states, but also the energy of the ground state alters due to strong coupling to the electromagnetic field. This change occurs even in the absence of light (due to the vacuum field); therefore, a manipulation of chemical reactivity and thermodynamics is possible by placing molecules inside an optical cavity, even without applying an external electromagnetic field. The shift of the ground level of the coupled system was first predicted (planar microcavity–intersubband transition of a semiconductor)^73^ and later observed (LC resonator magnectically coupled to a superconducting qubit)^200^ for systems in the ultrastrong coupling regime. For organic molecules, the first experimental observation of the modification of ground state thermodynamics happened in 2013.^176^ The thermodynamics of a planar cavity-molecules coupled system was studied by regarding the system as an equilibrium state between coupled (C) and uncoupled (U) molecules. Based on the fluorescence and absorption spectra, the equilibrium constant (*K* = [C]/[U]) of the system could be estimated. The standard Gibbs free energy difference (Δ*G*_C_) between the ground states of coupled and uncoupled molecules was further obtained using

**Fig. 8:**
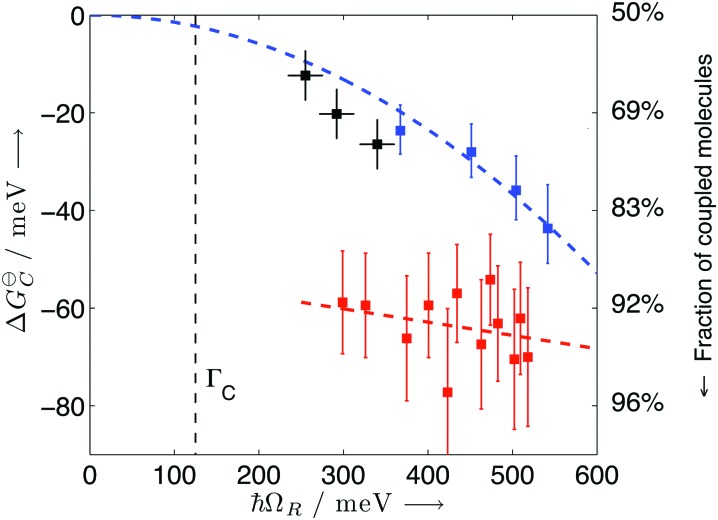
Standard Gibb's free energy and the fraction of coupled molecules as a function of Rabi splitting. The black, blue and red points represent data of TDBC, BDAB (5-(4-(dibutylamino)benzylidene)pyrimidine-2,4,6(1*H*,3*H*,5*H*)-trione) and merocyanine, respectively. The curves are linear or quadratic fits of the data points. The correspondence with the proportion of coupled molecules is given on the right side. Reproduced from ref. 176 with permission from John Wiley and Sons, copyright 2013.

23 Δ*G*θC = –*k*_B_*T* ln *K*

where *k*_B_ and *T* are Boltzmann's constant and temperature, respectively. By plotting Δ*G*_C_ against the Rabi splitting (Fig. 8), a direct relationship between the Rabi splitting and the ground state energy difference between the coupled and uncoupled molecules was confirmed. The temperature effects on systems with different coupling strengths were further investigated, showing changes in the standard entropy Δ*S*_C_ and the standard enthalpy Δ*H*_C_ associated with the coupling to the vacuum field through
24

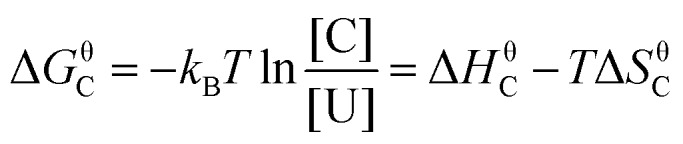

It was found that the entropy (Δ*S*_C_) is positive and dominates the final Δ*G*_C_ at room temperature, while the enthalpy (Δ*H*_C_) is extremely small. The positive entropy going from uncoupled states to coupled states suggests that the driving force for going into the strong coupling regime is the delocalization effect. It is worth noting that the fraction of the coupled molecules in these experimental systems was over 60%. This large attainable fraction of coupled molecules opens the door to tune molecular and material properties in the bulk. Furthermore, it was shown that the larger the Rabi splitting the larger the free energy difference between the coupled and uncoupled ground states. A free energy difference between the coupled and uncoupled ground states of 70 meV (7 kJ mol^–1^) was measured, thus large enough to affect the chemical equilibrium.

Many theoretical studies have been devoted to investigate the effects on molecules’ ground state under strong or ultrastrong coupling.^80^,^187^,^188^,^201^ Especially in the ultrastrong coupling regime, the rotating wave approximation for the molecule–light interaction becomes invalid. Instead of treating molecules as simple two-level systems, in 2015, Feist *et al.* investigated a first-principles model that fully takes into account both electronic and nuclear degrees of freedom.^188^ They and later others have found that some molecular properties could be affected by the collective coupling strength (ℏ*Ω*_R_), *e.g.* the energy shift of the ground-state. However, the effects on other properties such as bond length only depend on the single-molecule coupling *g*_0_ which is very small.^188^,^202^ Later, a similar conclusion was also reported with a model system of many molecules coupled to a surface-plasmon field.^80^ Thus, it seems that the prospects of ultrastrong coupling to change ground-state chemical reactions by directly affecting the bond-length are limited.

Although bond length modifications of molecules are small even in the ultrastrong coupling regime, the chemical energy landscape in the ground state can be manipulated by strong coupling to the vacuum electromagnetic field and holds promise to affect ground-state chemical reactions. The larger the Rabi splitting, the lower the free energy of the coupled state relative to the uncoupled state. Especially when the Rabi splitting accounts for a large ratio of the coupled transition energy, strong coupling will pose a non-trivial effect on the ground state energy landscape, making changes in chemical reaction rates and yields possible.

### 4.4 The effect of strong coupling on intermolecular interactions

**Fig. 9:**
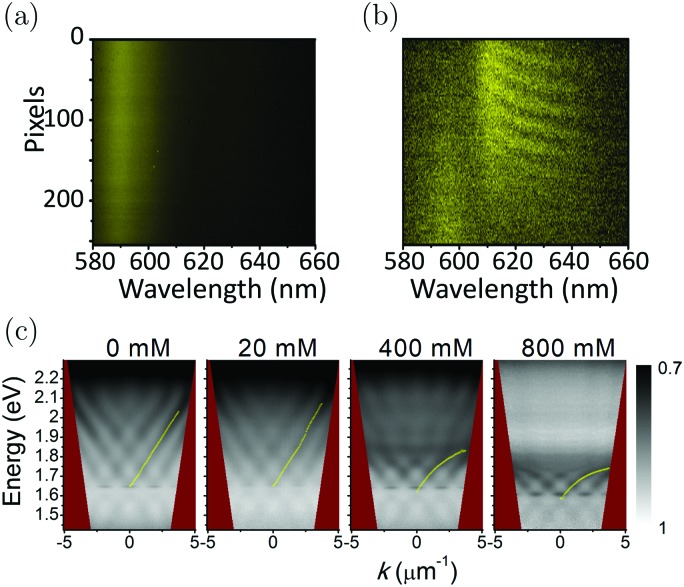
(a) Emission pattern for a TDBC layer on a glass substrate and (b) interference pattern for a TDBC layer on top of a flat silver layer. Reproduced from ref. 204 with permission from American Physical Society, copyright 2012. (c) Interference pattern with white areas representing the transmission maximum using different concentrations of DiD inside a polymer layer. Reproduced from ref. 205 with permission from American Physical Society, copyright 2014.

Excitons and charge carriers in films of organic molecules localize at individual molecules because of the strong exciton binding energy. At room temperature, charge carriers/excitons are usually transported between molecules through the so-called “hopping mechanism”—the charge carrier “jumps” from one molecule to another.^203^ In contrast, as a hybrid state of photon and exciton, the polariton inherits properties from the photon and delocalizes over the length scale of light (several hundreds of nanometers), corresponding to ∼10^5^ molecules. Compared to the localized exciton, the transport of polaritons across organic systems is therefore expected to be much more efficient and relies less on the microscopic structure of the system. Thus, strong exciton–photon coupling has the prospect of increasing the transport efficiency of excitons and charge carriers in organic systems. As already briefly mentioned, the spatial coherent emission over a macroscopic distance from a J-aggregated dye film induced by strong coupling was first observed in 2012.^204^ The diffusion and the spatial coherence of the emission of TDBC on a silver substrate were investigated using Young-type interferometric experiments. When in the strong coupling regime, a clear interference pattern (Fig. 9b) was observed between emitters separated by several microns, suggesting macroscopic in-phase emission. The coherence is absent in systems in the weak-coupling regime or in the absence of a plasmonic field (Fig. 9a), demonstrating the key role of the hybridization of the molecules with the plasmon. In another study, the behavior of the coherence of emission at the crossover from weak to strong coupling was examined. A system having a periodic silver nanoparticle array covered by DiD (1,1′-dioctadecyl-3,3,3′,3′-tetramethylindodicarbocyanine 4-chlorobenzenesulfonate) in a PMMA matrix was used. The change from weak to strong coupling regime was controlled by increasing the concentration of DiD inside the polymer matrix. In dispersion, the low concentration samples (weak coupling regime) showed a linear increase of the energy when increasing the in-plane wave vector *k*_‖_. However, at higher concentrations (strong coupling regime) the dispersion showed a bending and an anticrossing at the energy of the absorption maximum of DiD. The spatial coherence was investigated by inserting a double slit in the position of the image plane of the sample. For the sample in the strong coupling regime, the interference pattern showed the same bending as for the dispersion plot, and thus the interference fringes can be interpreted as replicas of the dispersion (Fig. 9c).^205^ By treating the exciton–vibrational coupling and exciton–photon coupling on an equal footing, it was later shown theoretically that the optical microcavities can enhance the exciton coherence length and eliminate vibronic coupling in J-aggregates.^206^

A direct observation of long-range (over several microns) transport of cavity polaritons in J-aggregates by ultrafast time-resolved microscopy was reported in 2018.^207^ It was shown that strong coupling between molecules and a cavity field induces long-range transport of excitons in the molecular system, and a propagation over several microns was observed. The propagation velocity of polaritons was found to be surprisingly slow (0.2–0.4 μm ps^–1^) as compared to the value predicted by polariton dispersion, suggesting that the transport of cavity polaritons in organic microcavities is not purely ballistic. The discrepancy is larger when the excitonic component of the polaritons is higher, which indicates that the relatively slow polariton migration originates from the disorder of the molecular system.

As for the cavity-enhanced transport of excitons, two groups reported the theoretical aspects of polariton transport in organic systems separately in 2015.^208^,^209^ The exciton-type transport in certain materials was found to be modified by strong coupling to the electromagnetic vacuum. A large increase in the transport for excitonic wave packets through a cavity was reported, as well as enhancement of steady-state exciton currents under incoherent pumping. The delocalized polariton modes were shown to be responsible for the exciton transport enhancement. Thus, strong coupling can help overcome the exponential suppression of exciton transport caused by disorder and other imperfections in a molecular system. These theoretical and experimental results have clearly shown that the migration of cavity polaritons in organic systems can be long-range and extend to the micrometer scale. This cavity-enhanced exciton/charge transport of organic systems is promising to improve the relatively poor efficiency of exciton/charge transport in organic materials. It holds promise to boost many applications including organic electronics and catalysis, which critically rely on the exciton/charge transport in the organic components.

**Fig. 10:**
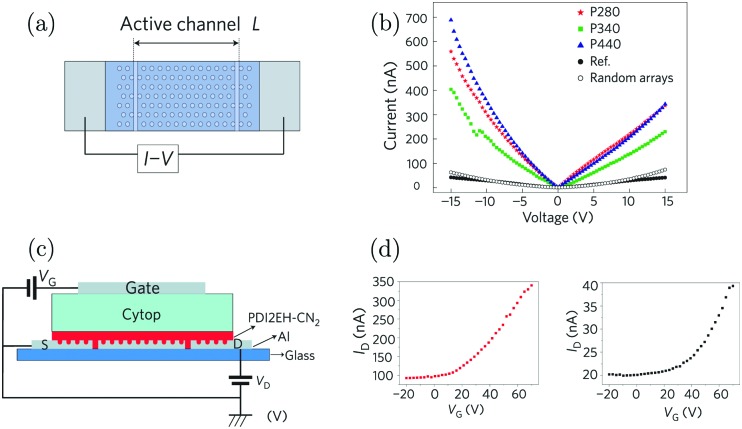
(a) Illustration of the configuration used to measure conductivity. The active materials were deposited on top of the Ag film with a hexagonal array. The current was measured through the electrodes at the end. (b) *I*–*V* curves as a function of systems in strong coupling regimes (blue, red and green) with metallic films with different periodic arrays. The black circles represent the data from the reference sample. (c) Three-terminal gating device geometry (transistor) used in the study. (d) Transfer curves of the transistors in the strong (left) and weak (right) coupling regimes. Reproduced from ref. 210 with permission from Springer Nature, copyright 2015.

Orgiu *et al.* reported the first experimental realization of enhancement of conductivity in the strong coupling regime in 2015.^210^ The conductivity of organic semiconductors by strong coupling to surface plasmons on metal hole arrays was enhanced. The conductivity and carrier mobility of two diimides were reported to increase by one order of magnitude following strong exciton–photon coupling. In the study, Ag or Al films with a periodic hexagonal array of holes were used (Fig. 10a). The surface plasmon resonance of the metal film is a function of hole periodicity, which was tuned to couple or decouple with the electronic transition of the diimides deposited directly on top. The conductivity of thin films of the organic semiconductor was evaluated by recording current–voltage (*I*–*V*) curves. The current was shown to increase by over one order of magnitude when the organic semiconductor was strongly coupled with a surface plasmon mode of the underlying metal hole array (Fig. 10b). Control experiments with no holes or random arrays of holes were also performed, showing no current increase. To further assess the electronic properties of the coupled system, a field-effect transistor structure was integrated together with the surface plasmon metal film and the charge carrier mobility was found to increase by an order of magnitude when the system was on-resonance (Fig. 10c and d). Thus, the increased carrier mobility in the coupled system accounts for most of the enhanced conductivity. However, the authors also noticed that the conductivity of thin films of similar molecules had no obvious enhancement effect, despite being strongly coupled, indicating that the disorder (thus smoothness of the energy landscape) in the organic system played a vital role in the charge transport even in the strong coupling regime. Later in 2017, Pupillo *et al.* theoretically demonstrated that light–matter coupling can lead to an enhancement of charge conductivity in a mesoscopic one-dimensional system.^211^ Importantly, they showed that the charge conductivity enhancement can reach orders of magnitude under experimentally realistic conditions.

These experiments clearly show that it is possible to utilize strong coupling to increase the conductivity of organic semiconductors, if the optical properties of the molecules are suitable for strong exciton–photon coupling. Other resonators like metal or dielectric Fabry–Pérot cavities could also potentially be integrated with organic systems to enhance their electrical performance, although no experimental demonstration of this kind of system has been reported yet. Expanding to other types of cavities will allow studying the enhancement effect in more detail, and show the generality of the concept using more straightforward cavity structures.

### 4.5 The effect of strong coupling on energy transfer

**Fig. 11:**
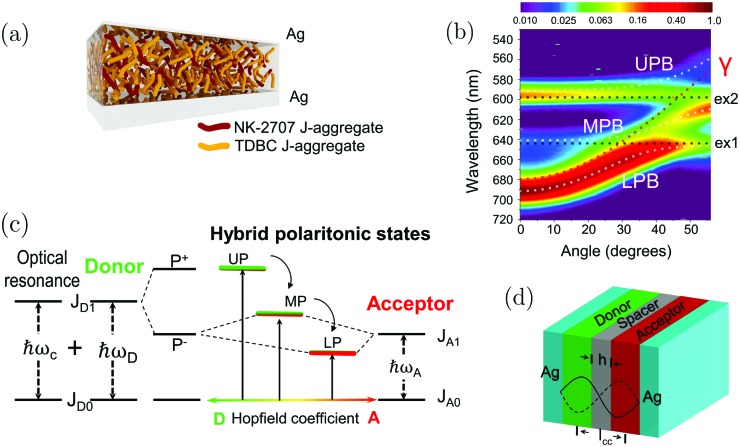
(a) The cavity structure used in ref. 203. (b) The angle-dependent photoluminescence from the microcavity. It clearly shows three polaritonic states, the upper, the middle and the lower polariton branches. Reproduced from ref. 212 with permission from Springer Nature, copyright 2014. (c) Energy diagram showing the cascade of energy levels formed when the donor and acceptor molecules are strongly coupled with the cavity photon. (d) The cavity structure adopted in ref. 205, showing the inert polymer spacer inserted between the donor and the acceptor molecules. Reproduced from ref. 214 with permission from John Wiley and Sons, copyright 2017.

The reports above deal mainly with cases in which a transition in one kind of molecule is strongly coupled to the cavity mode, thus forming two polaritonic states. When different species, *e.g.* a donor and an acceptor molecule, simultaneously couple to the same cavity mode and undergo optically driven mixing, a cascade of hybrid polaritonic states is formed. These quantum mechanically entangled hybrid states provide an effective path for energy transfer between molecules far apart to occur. The first realization of polariton enhanced energy transfer was reported in 2014.^212^ Two different J-aggregates (TDBC and NK-2707) were mixed in a supporting matrix and sandwiched into a cavity, supporting a confined 3*λ*/2 mode (Fig. 11a). The length scale of phase separation was sufficiently large to prevent direct dipole–dipole interactions and thus direct energy transfer between the two molecules. Angle-dependent photoluminescence (PL) emission revealed three branches (Fig. 11b), an upper (UPB), a middle (MPB) and a lower polariton branch (LPB). Analyzing these three branches using a coupled oscillator model, there is a clear mixing between the optical transitions of the two molecules. Moreover, the population of polaritons in the LPB is significantly greater than in either the UPB or MPB at all angles, providing qualitative evidence for an efficient energy relaxation pathway that depopulates both the UPB and MPB. Based on the photoluminescence study, it was argued that the MPB presents a channel that facilitated energy transfer between the two molecules. The energy transfer efficiency is dependent on the exciton mixing present in the MPB. Such a process represents a new non-radiative energy-transfer mechanism that can transfer energy between molecules separated by length scales much larger than the Förster transfer radius. Later, similar results showing a seven times increase of energy transfer between two different dyes when strongly coupled to the vacuum field were demonstrated.^213^ In the following report, the polariton enhanced energy transfer was further confirmed even for spatially separated (by an inert PMMA layer) donor and acceptor cyanine dyes (Fig. 11d).^214^ Because of the energy cascade of the delocalized polaritonic states (Fig. 11c), the transfer efficiency at donor–acceptor distances of over 100 nm can approach 37% (far longer than the distances reached with the FRET mechanism). It is worth noting that the energy transfer process is independent of the distance as long as the coupling strength is maintained, which is consistent with the entangled and delocalized nature of the polaritonic states. Later, it was found that the cavity detuning also plays an important role in the energy transfer efficiency and negative detuning can lead to a more efficient transfer to the lower polaritons.^215^

Two different groups reported the theoretical aspects of polariton assisted energy transfer almost at the same time. The Garcia-Vidal group adopted a Fabry–Pérot cavity, which is exactly the same configuration as used in the earlier experimental reports.^216^ The phenomenon was addressed numerically by means of the Bloch–Redfield theory, allowing the effect of complex vibrational reservoirs characteristic of organic molecules to be reproduced. The delocalized middle polariton was revealed to play a key role as the delocalized intermediary in the energy transfer process. The major conclusion was that the polariton assisted energy transfer is controlled by the coupling strength and does not depend on the particular arrangement of the molecules inside the cavity or the electromagnetic mode spatial profile. They also provide specific recipes to optimize the energy transfer process based on their analytical approach. Yuen-Zhou *et al.* presented a comprehensive theory of polariton-assisted remote energy transfer (PARET) based on the strong-coupling of donor and/or acceptor chromophores to surface plasmons.^217^ Application of the theory demonstrates that PARET up to one micrometer is indeed possible. The conditions were highlighted under which coherence enhances or deteriorates the PARET processes. Particularly, strong-coupling to acceptors can shift energy levels in a way that energy transfer can happen from an acceptor to a donor molecule, thus resulting in a chromophore role-reversal. Both theories demonstrate the potential for strong coupling to control energy transfer in organic systems with both optical and plasmonic cavities.

To conclude, polariton-assisted energy transfer has been clearly demonstrated both experimentally and theoretically. The delocalized nature of the formed polaritons enables an efficient and ultrafast energy-transfer pathway between donor and acceptor molecules. As long as the strong coupling regime is reached, the efficiency of energy transfer does not depend on the physical separation between the molecules inside the cavity. Thus, the delocalized polariton allows energy transfer to occur at much larger distances as compared to the Förster dipole–dipole interaction or the Dexter electronic exchange mechanisms. The possibility of polaritons to transfer energy between molecules at considerably larger distances than before was confirmed, which has important implications for molecular energy transfer related processes, such as light-harvesting and organic electronics.

## 5. Beyond polariton chemistry

The promise of engineering molecular properties with light has so far led to a plethora of potential applications. Especially novel spectroscopy techniques based on hybrid molecular systems having modified optical responses could emerge.^201^ For example, the Raman scattering cross-section area of vibrational groups demonstrates orders of magnitude enhancement in the strong coupling regime,^193^,^218^,^219^ as well as second and third harmonic generation.^220^,^221^ Furthermore, organic light emitting devices in the strong coupling regime exhibit interesting properties, such as directed emission and extended responsivity.^83^,^88^,^90^,^222^–^226^ Recently, Shi *et al.* demonstrated a many-fold increase in the photon to current conversion efficiency and an increase in the quantum efficiency of water splitting in the strong coupling regime.^227^ Other aspects including coherent light harvesting^228^ and polariton-assisted singlet fission^229^ were also predicted theoretically. The following sections cover some recent advances and applications of strong light–matter interactions such as polariton lasing and room temperature Bose–Einstein condensates. Strong light–matter interactions of two dimensional materials and carbon nanotubes, of high relevance in optoelectronic devices, are also covered.

### 5.1 Bose–Einstein condensation and lasing

A Bose–Einstein condensate (BEC) is an aggregation state that has a high density of bosonic particles in the lowest quantum state, leading to spatially overlapping wave functions of the particles.^230^ The overlapping wave functions give rise to macroscopic quantum phenomena such as interference between the wave functions of the particles. Polaritons formed in the strong coupling regime can be used to produce room temperature BECs and lasing systems. Due to their low effective mass and high binding energy of organic excitons, polaritons have the possibility to produce BECs at higher temperatures as compared to atoms, which require cryogenic conditions. Kasprazak *et al.* demonstrated the first polariton BEC using a CdTe-based microcavity.^20^ Inorganic based exciton–polariton BECs have since been used to demonstrate thermal equilibrium below the threshold,^20^,^21^ threshold corresponding to onset of degeneracy,^19^–^21^ narrowing of the linewidth,^20^,^21^ long-range spatial coherence,^20^,^21^,^231^ temporal coherence,^20^,^232^ spontaneous polarization^233^ and polariton accumulated coherence.^234^ However, a controversial issue is whether the exciton–polariton BECs are BECs or polariton lasers.^235^–^238^

Polariton lasing using organic molecules was demonstrated before organic molecule based polariton condensates. In the first example of polariton lasing, an anthracene single crystal placed between two DBR mirrors was used.^141^ At room temperature the lasing threshold was at *P*_th_ = 320 μJ cm^–2^, a lower value as compared to the best-case estimates of the threshold (430 μJ cm^–2^) for conventional photonic lasing using this system. The characteristic properties after reaching the threshold were spatial modulation of the pump spot, a change in the polariton distribution function, a collapse of the emission lifetime and spectral narrowing. In another study, a plasmonic lattice was used instead of an optical cavity.^239^ As emitter a perylene bisimide derivative inside a PMMA matrix, spin-coated on top of the plasmonic structures, was used. It was found that the lasing threshold of the system depends on the concentration of the dye inside the polymer matrix; the higher the concentration the lower the lasing threshold value. Polaritonic lasing has also been demonstrated for a hybrid organic–inorganic system inside an optical cavity.^240^ A J-aggregate forming cyanine dye and a GaAs quantum well were hybridized to a cavity mode. The energy exchange between the organic and inorganic layers was efficient and polariton lasing was demonstrated up to a temperature of 200 K with a threshold of 16.1 μJ cm^–2^. These findings show that it is possible to achieve polariton lasers using organic molecules, and that lasing starts at very low thresholds.

**Fig. 12:**
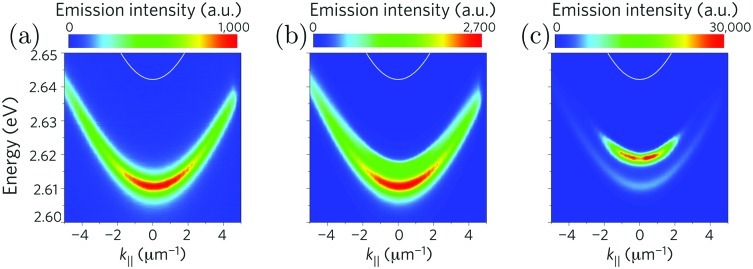
Emission from the lower polariton (a) below the threshold, (b) near the threshold and (c) above the threshold measured by momentum-resolved time-integrated spectroscopy. A blueshift of the emission is observed in (b and c), and the emission intensity increases nonlinearly. Reproduced from ref. 23 with permission from Springer Nature, copyright 2013.

The conditions for polariton condensation in organic cavities were theoretically predicted in 2012.^241^ Around one year later, two different groups demonstrated organic based exciton–polariton BECs experimentally. Plumhof *et al.* used a poly(*p*-phenylene) derivative (MeLPPP) sandwiched between two DBR mirrors.^23^ The optically active polymer layer was prepared by spin coating, leading to an amorphous film. The condensate was observed when the excitation density was increased to the threshold density of *P*_th_ ∼ 500 ± 200 μJ cm^–2^ (8 ps long). Kéna-Cohen *et al.* used the organic semiconductor TDAF (2,7-bis[9,9-di(4-methylphenyl)-fluoren-2-yl]-9,9-di(4-methylphenyl)fluorine) in a microcavity.^242^ The optical properties of the condensates differ substantially from those of the polaritons. The emission peak at *k*_‖_ = 0 was blue shifted, indicating a repulsive interaction between polaritons, and a nonlinear increase in emission intensity as a function of pumping intensity was observed as well as a sudden decrease in bandwidth (Fig. 12a–c). In addition, the emission was polarized like the pump beam and showed a long-range phase coherence, which was proven by the use of a Michelson interferometer. In a further investigation, the same changes in the optical properties in a polariton condensate were observed.^24^ The difference between these studies was that the small molecule BODIPY-Br (emitting at 565 nm) in a polystyrene matrix was now used instead of the polymer MeLPPP (emitting at 450 nm). These findings indicate that many different dyes can be used to form polariton condensates, spanning condensates in the visible to near infrared regime of the electromagnetic spectrum. The possibility to reach room temperature exotic matter phases offers a way to explore new chemistry.^243^ Furthermore, electrically pumped polariton lasers open the door to engineer devices being orders of magnitude more energy efficient due to the low lasing threshold.^244^,^245^

### 5.2 SC using 1D and 2D materials

One dimensional (*e.g.* carbon nanotubes) and two dimensional (*e.g.* graphene, perovskites or transition metal dichalcogenides) materials generally have strong interactions with light. Some of them have been identified as semiconductors with band gaps in the infrared and visible spectral region, making them suitable for a variety of applications in optics and optoelectronics.^246^ Because of the strong geometrical confinement and the weak dielectric screening, different types of excitons can be formed, including optically allowed and forbidden dark excitons, and excitons with coupled spin and valley degrees of freedom. Strong coupling of these materials will extend their applications as polaritonic optoelectronics and lead to novel phenomena related to exciton physics in condensed matter.^247^

**Fig. 13:**
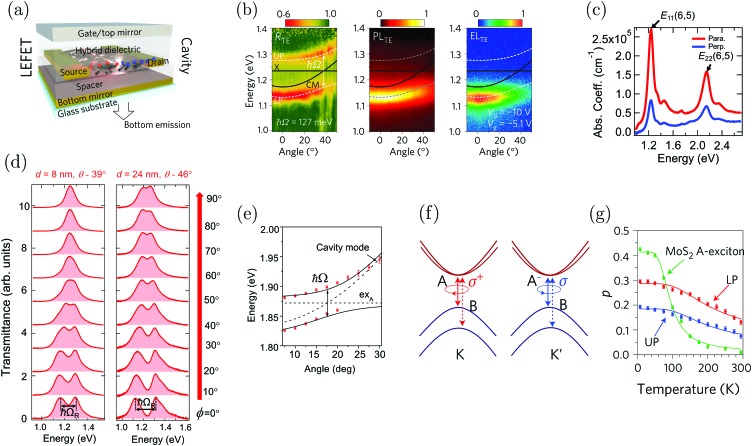
(a) Schematic geometry of a bottom-contact/top gate light emitting field-effect transistor integrated into an optical cavity. (b) Angle- and spectrally resolved reflectivity (left), photoluminescence (middle) and electroluminescence (right) of the channel area in the device. The emission from both the photoluminescence and electroluminescence agrees with the reflectivity spectra and can be fitted well using a coupled oscillator model. Reproduced from ref. 255 with permission from Springer Nature, copyright 2017. (c) The polarization-dependent absorption spectra of an aligned SWCNT film, showing the first and second interband exciton peaks, *E*_11_ and *E*_22_. (d) Experimental transmittance spectra at zero detuning for various polarization angles (from 0° to 90°) for a device working in the *E*_11_ region using SWCNT films having different thicknesses (*d*). Reproduced from ref. 58 with permission from Springer Nature, copyright 2018. (e) Dispersion relation extracted from the angle-resolved reflectivity spectra of the cavity based on the MoS_2_ monolayer. The two black solid curves correspond to theoretical fits of the polariton branches using a coupled oscillator model. Reproduced from ref. 261 with permission from Springer Nature, copyright 2015. (f) Schematic showing the valley-dependent optical selection rules at inequivalent *K* and *K*′ valleys at the edges of the Brillouin zone. (g) Emission polarization for bare excitons (MoS_2_ excitons), upper polariton (UP) and lower polariton (LP) branches as a function of temperature. Reproduced from ref. 271 with permission from Springer Nature, copyright 2017.

Single-walled carbon nanotubes (SWCNTs) usually show narrow linewidth emission (full width at half maximum, FWHM, ∼20 meV),^248^ a large oscillator strength^249^ and high exciton binding energies.^250^ Furthermore, semiconducting SWCNTs possess extremely high charge carrier mobility^251^ and emit in the near infrared.^252^ Strong exciton–photon coupling of SWCNTs was realized in metallic Fabry–Pérot microcavities as well as plasmonic cavities of a periodic gold nanodisk array in 2016.^253^,^254^ A Rabi splitting over 110 meV was achieved, and both angle-dependent reflectivity and photoluminescence spectra showed clear anti-crossing behaviour and agreed well with simulations based on the coupled oscillator model and the transfer-matrix model. In the following paper, electrically pumped near-infrared exciton–polariton emission was demonstrated using a SWCNT-based ambipolar light-emitting field-effect transistor that was embedded in a metallic Fabry–Pérot microcavity (Fig. 13a).^255^ The dispersive behaviour of the lower polariton agrees well with each other in the angle- and spectrally resolved reflectivity, photoluminescence and electroluminescence (Fig. 13b). The electrically pumped polariton emission and the efficient polariton relaxation (to the P^–^ state) of the system were realized even at very high current densities. The narrow-band polariton electroluminescence from 1060 nm to 1530 nm could be achieved by simple adjustments in the cavity thickness without modifying the active materials inside. The coupling strength in the light-emitting field-effect transistor could be reversibly tuned by unipolar charge carrier accumulation, with the resulting reduction in the oscillator strength of the SWCNTs. Charged excitons are usually termed trions and consist of a neutral exciton and an additional hole or electron. Trion–polariton formation in SWCNTs has also been observed in a later report, which has implications for the realization of polaritonic charge transport.^256^ In a more recent work, aligned SWCNTs inside a Fabry–Pérot microcavity were tuned from the weak to the strong and ultrastrong coupling regimes through simple polarization rotation, taking advantage of the strong anisotropic absorption of SWCNTs (Fig. 13c and d).^58^ Tuning of the light–matter coupling strength was shown both for the first interband exciton–polaritons (*E*_11_) in the near-infrared range and for the second interband exciton–polaritons in the visible range (*E*_22_) (Fig. 13c). The maximum Rabi splitting observed was 329 meV (for the second interband exciton–polaritons), the highest value ever reported for a Wannier exciton. Continuous mapping of the polariton dispersion surfaces revealed two pairs of exception points, where the lower and upper polaritons meet in energy. The two exception points are bounded together with an equal-energy arc, forming a line in momentum space where the system is not in the strong coupling regime any more. These anisotropic SWCNT-based polaritons in the strong and ultrastrong coupling regime can lead to novel phenomena and devices, including room-temperature polariton condensation and 1D laser diodes.

Two dimensional materials, especially transition-metal dichalcogenides (TMD) which possess a direct bandgap, have emerged as a new class of materials that demonstrate strong interactions with light.^246^,^257^–^260^ TMD is a group of naturally abundant materials having an empirical formula of MX_2_, where M is a transition-metal from group VI (M = Mo, W) and X is a chalcogen (X = S, Se, Te). These atoms form a hexagonally coordinated structure with the metal atom layer sandwiched between top and bottom chalcogen layers, leading to a trigonal prismatic crystal structure. One of the most intriguing features of TMDs is the emergence of fundamentally distinct electrical and optoelectrical properties when comparing the bulk material and the two-dimensional limit (monolayer). TMDs provide a mostly disorder-free two-dimensional system, while keeping a reasonably large exciton binding energy, enabling the possibility to observe non-linear polariton interactions at room temperature as well as new features, such as valley polarization, introduced by TMD excitons.

The first report of strong coupling of two-dimensional exciton–polaritons was based on a MoS_2_ monolayer embedded in a dielectric microcavity.^261^ The angle-resolved reflectivity and the photoluminescence from the microcavity clearly exhibited an anti-cross behaviour and a Rabi splitting of 46 meV was obtained based on fitting to a coupled oscillator model (Fig. 13e). The spectral half width for excitons and cavity photons was 30 and 9 meV respectively, thus satisfying the criteria of emergence into the strong coupling regime (eqn (14)). Ever since this work, strong coupling based on different TMDs and cavity systems has been reported, *e.g.* MoSe_2_/h-BN quantum well-dielectric microcavity,^262^ MoS_2_-plasmonic arrays,^263^ WS_2_-metallic planar cavity,^264^ and WS_2_/WSe_2_-plasmonic nanoparticles.^127^,^265^–^267^

Beyond just reaching the strong coupling regime, novel intriguing phenomena related to TMDs strongly coupled to cavity photons have been studied, including polariton fluids,^268^ electric field gating effects,^269^ second harmonic generation,^270^ and valley polarization.^271^–^275^ In particular, 2D excitons in TMD monolayers showed spin–valley coupling arising from the combination of the unique properties of the hexagonal crystal.^276^,^277^ The peculiar crystal structure of TMDs introduces a degeneracy in the exciton energy: opposite spins are unambiguously associated to different valleys in the momentum space, allowing direct optical initialization with opposite circular polarization of the exciton spin in the non-equivalent valleys (Fig. 13f). However, the need for cryogenic operation limits the practical applications. In 2017, three different groups reported simultaneously in Nature Photonics that spin–valley locking persists in the strong coupling regime at room temperature, showing that valley polaritons can be coherently excited by helical cavity fields and can spatially coexist in separate regions of momentum space.^271^–^273^ The interplay of intervalley depolarization and cavity-modified exciton dynamics in the high-cooperativity regime leads to valley-polarized exciton–polaritons to persist at room temperature, distinct from the vanishing polarization in bare monolayers (Fig. 13g). The realization of valley polaritons in 2D semiconductor microcavities presents the first step towards engineering valley-polaritonic devices.^246^

Two-dimensional materials are a promising class of materials, whose properties can be further tuned by strong exciton–photon coupling, as to enhance and extend their applications. Exciting phenomena have already been achieved including the above-mentioned valley-polarized coupling at room temperature. There are other intriguing phenomena and applications waiting to be explored, such as lasing and controlled polariton flow.

## 6. Future perspectives and concluding remarks

Strong exciton–photon coupling started as a subfield within atomic physics. About 25 years ago, the solid state physics community started using the concept on inorganic semiconductors. 20 years ago, organic molecules were strongly coupled to light for the first time. 10 years ago, the idea emerged that strong coupling can be used to change molecular properties, and thus to change the energy landscape and the energy transfer/relaxation pathways of molecules, which can be used within broad areas ranging from organic chemistry to cold atom physics. The big breakthrough from an application perspective is yet to be realized, but to foresee, we expect the first application using strong exciton–photon coupling (using organic molecules) to take advantage of either the enhanced charge conductivity or the ability to channel excitation energy. Channeling of excitation energy can for instance be due to a rearrangement of energy levels. It has been shown that it is possible to increase the rate of reversed intersystem crossing due to lowering of a singlet state (*i.e.* lower polariton) without modifying a triplet state located relatively close in energy. This is of practical importance within organic electronics, where excited singlet states are much preferred over triplet states. Channeling of excitation energy can also be the energy dispersion dispersivity of the lower polariton, giving a driving force for polariton condensation and lasing. This allows for collimated and coherent emission, which are of practical importance for both lasers and general light-emitting applications. Furthermore, strong coupling is not only limited to electronic transition. Coupling vibrational bands to light can open the possibility to a new approach within chemistry. Recent breakthroughs in the field have shown that strong coupling to a vibrational transition can induce site-selectivity in a chemical reaction^278^ and order of magnitude increase in the reaction rate of hydrolysis.^199^

For in-depth understanding of the strong coupling phenomena, a solid theoretical framework is of importance. The coupled oscillator model has served the community well, and will continue to be used to provide information on coupling strength and Hopfield coefficients, two of the most important parameters when characterizing a strongly coupled system. However, organic molecules are vastly more complex than atoms. Theoretical description of the excited state dynamics of a single organic molecule in a cavity is challenging, since such system cannot be described with only quantum chemistry and quantum optics concepts. It is thus naïve to expect that the coupled harmonic oscillator model will accurately simulate excited state processes in a strongly coupled system containing organic molecules. The new development of quantum electrodynamic DFT and cavity Born–Oppenheimer approximation is therefore very promising and can help to design novel experiments.^279^–^286^ Having access to both robust and simple theoretical tools (the coupled oscillator model) and *ab initio* theory (QEDFT) where intricate questions can be asked and answered is vital for future progress of the research field.

As seen throughout this review article, the exciting research field of strong light–matter coupling is still in its infancy, but today researchers from various disciplines are starting to set their eyes on the concept. This influx of new viewpoints is an insurance of a continuation of the rapid development seen within the field, but also offers the prospect that the concept may be used in fundamentally different research areas than conceivable today, such as sensing^287^,^288^ and chemistry quantum information.^289^ It is only the imagination that will put a limit on where strong light–matter coupling can be exploited: why not do photochemistry without the need of light?

## Conflicts of interest

There are no conflicts to declare.
