# Correction: Strong light–matter interactions: a new direction within chemistry

**DOI:** 10.1039/d0cs90077j

**Published:** 2020-09-08

**Authors:** Manuel Hertzog, Mao Wang, Jürgen Mony, Karl Börjesson

**Affiliations:** University of Gothenburg, Department of Chemistry and Molecular Biology Kemigården 4 41296 Gothenburg Sweden karl.borjesson@gu.se

## Abstract

Correction for ‘Strong light–matter interactions: a new direction within chemistry’ by Manuel Hertzog *et al.*, *Chem. Soc. Rev.*, 2019, **48**, 937–961, DOI: 10.1039/C8CS00193F.

The authors regret that eqn (16) was missing a factor of ½. The corrected equation is
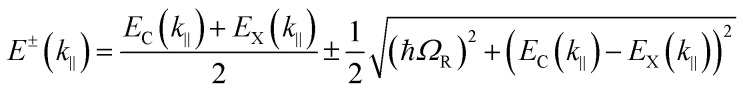


The Royal Society of Chemistry apologises for these errors and any consequent inconvenience to authors and readers.

## Supplementary Material

